# Novel xylose transporter Cs4130 expands the sugar uptake repertoire in recombinant *Saccharomyces cerevisiae* strains at high xylose concentrations

**DOI:** 10.1186/s13068-020-01782-0

**Published:** 2020-08-14

**Authors:** João Gabriel Ribeiro Bueno, Guilherme Borelli, Thamy Lívia Ribeiro Corrêa, Mateus Bernabe Fiamenghi, Juliana José, Murilo de Carvalho, Leandro Cristante de Oliveira, Gonçalo A. G. Pereira, Leandro Vieira dos Santos

**Affiliations:** 1 Brazilian Biorenewable National Laboratory (LNBR), Brazilian Center for Research in Energy and Materials (CNPEM), Campinas, São Paulo 13083-100 Brazil; 2 Genetics and Molecular Biology Graduate Program, Institute of Biology, University of Campinas (UNICAMP), Campinas, Brazil; 3 Brazilian Biosciences National Laboratory (LNBio), Brazilian Center for Research in Energy and Materials (CNPEM), Campinas, Sao Paulo 13083-970 Brazil; 4 Brazilian Synchrotron Light Laboratory (LNLS), Brazilian Center for Research in Energy and Materials (CNPEM), Campinas, Sao Paulo 13083-970 Brazil; 5 Department of Physics-Institute of Biosciences, Humanities and Exact Sciences, UNESP, São Paulo State University, São José do Rio Preto, São Paulo 15054-000 Brazil

**Keywords:** Xylose, Xylose transporter, Major facilitator superfamily, *Saccharomyces cerevisiae*, Pentose metabolism, Industrial biotechnology

## Abstract

**Background:**

The need to restructure the world’s energy matrix based on fossil fuels and mitigate greenhouse gas emissions stimulated the development of new biobased technologies for renewable energy. One promising and cleaner alternative is the use of second-generation (2G) fuels, produced from lignocellulosic biomass sugars. A major challenge on 2G technologies establishment is the inefficient assimilation of the five-carbon sugar xylose by engineered *Saccharomyces cerevisiae* strains, increasing fermentation time. The uptake of xylose across the plasma membrane is a critical limiting step and the budding yeast *S. cerevisiae* is not designed with a broad transport system and regulatory mechanisms to assimilate xylose in a wide range of concentrations present in 2G processes.

**Results:**

Assessing diverse microbiomes such as the digestive tract of plague insects and several decayed lignocellulosic biomasses, we isolated several yeast species capable of using xylose. Comparative fermentations selected the yeast *Candida sojae* as a potential source of high-affinity transporters. Comparative genomic analysis elects four potential xylose transporters whose properties were evaluated in the transporter null EBY.VW4000 strain carrying the xylose-utilizing pathway integrated into the genome. While the traditional xylose transporter Gxf1 allows an improved growth at lower concentrations (10 g/L), strains containing Cs3894 and Cs4130 show opposite responses with superior xylose uptake at higher concentrations (up to 50 g/L). Docking and normal mode analysis of Cs4130 and Gxf1 variants pointed out important residues related to xylose transport, identifying key differences regarding substrate translocation comparing both transporters.

**Conclusions:**

Considering that xylose concentrations in second-generation hydrolysates can reach high values in several designed processes, Cs4130 is a promising novel candidate for xylose uptake. Here, we demonstrate a novel eukaryotic molecular transporter protein that improves growth at high xylose concentrations and can be used as a promising target towards engineering efficient pentose utilization in yeast.

## Background

Concerns regarding the mitigation of climate change and global warming impacts have led to a commitment to restructure the energy matrix based on fossil fuels. One promising and cleaner energy alternative demands the development of sustainable advanced biofuels produced from renewable lignocellulosic biomass, also known as second-generation (2G) biofuels [1–4]. The conversion of plant biomass sugars into bioproducts requires the extraction and breakdown of the main macromolecular components from the recalcitrant structure of the plant cell wall. The complex architecture of plant cell wall contains cellulose (40–50%), hemicellulose (25–35%), and lignin (15–20%) as major constituents [5, 6]. Cellulose consists of chains of glucose, the most abundant sugar monomer in biomass, while the hemicellulosic fraction contains xylose, a five-carbon sugar, as the main component, being the second most abundant monomer in the lignocellulosic portion.

The conversion of biomass-derived sugars into different bioproducts requires the development of robust and high-yielding microbial platforms through synthetic biology approaches. The budding yeast *Saccharomyces cerevisiae* has been traditionally used as a eukaryotic platform model to design new routes and engineer metabolic fluxes towards desired high-value bioproducts, such as fuels, chemicals, food, feed, and pharmaceuticals [7, 8]. However, the C5 sugar xylose is not naturally metabolized by wild-type strains of *S. cerevisiae*, requiring systems metabolic engineering strategies to develop high-performance strains [9, 10]. Xylose catabolism in *S. cerevisiae* occurs through the integration of heterologous metabolic pathways associated with adaptive evolution strategies to reshape cellular metabolism and improve fermentation fitness [10–13]. Mutations related to fitness-enhanced phenotypes arise during evolution to alleviate metabolic bottlenecks and speed up xylose conversion [10, 12]. By using these combined approaches, it is possible to improve catabolic fluxes and engineer strains to assimilate xylose more efficiently. However, several limitations still need to be addressed to optimize biomass conversion, including the inefficient transport of xylose by *S. cerevisiae* strains.

The transport of sugars is mediated through transmembrane proteins, which perform the uptake of a broad range of substrates between extracellular and intracellular environments of the cell. Sugar transporters are found mainly in the major facilitator superfamily (MFS), spread in all branches of life [14–17]. The MFS members are divided into three classes: uniport, transports a single substrate; symporter, the transport of the substrate is usually coupled to an ion; and antiport, which transports two different substrates in opposite directions [14, 15, 18]. MFS transporters are branched in 74 subfamilies [19], some of them including the well-known xylose transporters *GAL2* [20–24], *HXT* [20–23], *GXF1* [21, 25, 26] and *GXS1* [27–29]. The budding yeast *S. cerevisiae* has a set of native hexose transporters (Hxt1p, Hxt2p, Hxt4p, Hxt5p, Hxt7p, and Gal2p) which can also import xylose, but with low affinity and strong glucose repression, limiting fermentation of mixed biomass sugars [30, 31]. *S. cerevisiae* endogenous transporters have a preference to assimilate glucose over xylose in industrial lignocellulosic hydrolysates, increasing the time of fermentation [32]. The expression of heterologous xylose transporters in engineered *S. cerevisiae* strains has already resulted in improved xylose assimilation rates. For example, the most well-described facilitator, Gxf1, isolated from *Candida intermedia*, improved transport kinetics and xylose utilization in recombinant xylose-fermenting *S. cerevisiae* by threefold [25, 26, 33–35]. The glucose/xylose-facilitator Gxf1 from *C. intermedia* is considered the most efficient transporter when compared to other known heterologous xylose transporters, such as Sut1, Xut1, Xut3 (*Scheffersomyces stipitis*), At5g59250 (*Arabidopsis thaliana*) and XylHP (*Debaryomyces hansenii*) [21, 26, 33]. Interestingly, the transport of xylose in Gxf1 seems to be concentration-dependent given that improvements in transport rates and growth were observed only at very low xylose concentrations (~ 4 g/L), while remained unchanged at higher concentrations [21, 25, 33].

The inefficient and unspecific xylose transport across the plasma membrane in *S. cerevisiae* is a major drawback to design microbial platforms for industrial biomass conversion [36]. Identifying superior transporter proteins and molecular mechanisms for improved xylose utilization through metabolic engineering is essential for the development of 2G biorefineries. In this work, we described a novel membrane transporter protein that improves growth in high xylose concentrations and can be used to engineer *S. cerevisiae* strains for efficient pentose conversion in biobased fuels and chemicals.

## Results

### Isolation and molecular identification of C5 yeasts

Only a small number of heterologous sugar transporters capable of enhancing xylose uptake in *S. cerevisiae* have been identified and characterized so far. In order to explore novel yeast C5 uptake systems, we accessed the microbiome of potential sources such as decayed energy cane and sugarcane, insect plagues isolated from these materials and also from corn crops (Additional file 1: Figure S1). A total of 300 yeast colonies were recovered from enrichment in xylose medium and molecularly identified based in D1/D2 domains and the ITS region. The consecutive batches using the xylose isolation medium were employed to enrich the population and select only strains capable of assimilating xylose more efficiently, resulting in only a few species from each material that dominated the population. The presumable wide diversity in populations colonizing the starting samples was probably severely reduced in response to a selective xylose-enriched environment. Additional file 2: Table S1 summarizes the 13 yeast species identified and source of isolation, confirming the dominance of few species in each material, resulting from adaptation and competition regarding the use of xylose as the sole carbon source.

### Comparative xylose fermentation

The enriched yeast culture libraries represent a potential source of novel specific transport systems for xylose. To discover novel potential gene donor candidates for heterologous expression of C5 transporters in *S. cerevisiae*, comparative fermentation assays using xylose as the sole carbon source were performed in order to measure fitness and xylose consumption rates. All species were able to consume xylose and produce xylitol preferably, with low concentrations of glycerol and ethanol also observed as fermentation byproducts. Xylitol was the main product observed due to a redox cofactor imbalance caused by a xylose reductase with a higher affinity to NADPH and a xylitol dehydrogenase NAD^+^ dependent, a pattern traditionally present in wild C5 yeasts. The kinetic parameters for fermentation assays are summarized in Additional file 2: Table S1. All yeast cells were able to consume xylose with a wide difference in consumption rates. After 54 h of experiment, starting with 20 g/L of xylose, the differences ranged from 28.5 to 100% of xylose consumed. The four yeasts from *Candida* genus presented the best performances regarding the assimilation of xylose with approximately 90% of the sugar consumed for *C. boidinii* and 100% for the others in the group. Except for *C. boidinii*, the other *Candida* yeasts presented the highest yields of xylitol with a maximum of ~ 0.67 g xylitol/g xylose produced by *C. sojae*. *Wickerhamomyces anomalus* also performed well in terms of xylitol production, with a yield of 0.54 g xylitol/g xylose. In YPX containing 30 g/L, under aerobic conditions, the *C. sojae* isolate consumed all the sugar in approximately 70 h, starting with a low cell density, producing xylitol as the main product followed by very low concentrations of ethanol and glycerol (Additional file 3: Figure S2). We hypothesized that, since *C. sojae* displayed a fast xylose consumption compared to the other strains, this isolate could be a promising source of novel xylose transporter-encoding genes for heterologous expression in *S. cerevisiae*.

### Genome mining of xylose transporter-encoding genes from *C. sojae*

**Fig. 1:**
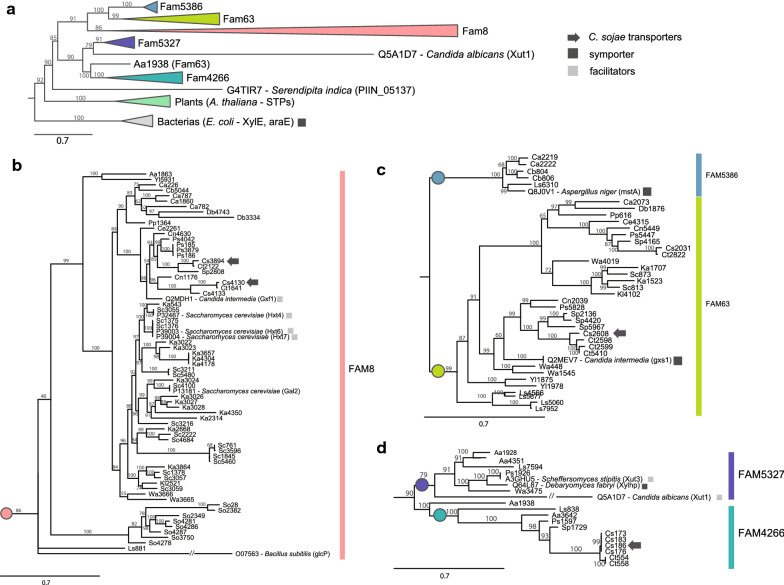
Phylogenetic inference for transporter gene families in yeasts. The gene tree was reconstructed using maximum likelihood method as implemented in IQtree, with branch support estimated using 1000 bootstrap replications. A collapsed version showing relationships among families and the known transporters for other fungi, plants and bacteria are shown in **a**. Expanded versions for each family of yeast transporters are presented in **b**–**d**. Already characterized xylose transporters are identified with squares indicating the transporter type. Dark arrows indicate *C. sojae* transporters

A set of known genes which encode microbial xylose transporters were retrieved from public databases and used as baits to screen putative promising transporters in the *C. sojae* genome and classify as symporter or facilitator. The transporter genes phylogeny indicate that all xylose transporters are homologous, i.e., have a unique ancestor gene (Fig. 1). Gene families attributed by OrthoMCL pipeline revealed as monophyletic groups in the whole gene tree, supporting that those families are homolog groups with independent evolutionary trajectories. The only exception was Aa1938, which was closer to Fam4266 than to the rest of Fam63, with whom it was clustered in OrthoMCL (Fig. 1a). Four of the five gene families had at least one known xylose transporter clustered in their clades allowing some inference on the phenotype of each family genes. Comparing the yeast transporter genes with other fungi, the mstA transporter from *Aspergillus niger* is in the family 5386 tree and grouped with a *Lipomyces starkeyi* transporter (Ls6310). This observed relationship is not expected considering the species’ phylogeny as *A. niger* is an *Ascomycete* from a different Class. In the same way, the bacterial transporter glcp from *Bacillus subtilis* clustered with family 8’s yeast transporters instead of with other bacterial transporters as would be expected. The incongruence among gene trees and the species phylogeny may arise from different factors, such as horizontal gene transfers, incomplete lineage sorting, and also similar selective pressures leading to sequence convergence.

Gene family expansions are easily recognized throughout the yeast transporter’s phylogeny (Fig. 1). In family 8, Cs3894 and Cs4130 appear as copies from a putative gene duplication in the base of *Pichia*, *Spathaspora* and *Candida* clade (Fig. 1b), where the first shares a subclade that follows the species’ phylogeny [37] and the second in a subclade exclusive of *C. sojae*, *C. tropicalis*, and *C. tanzawanensis*, putatively being lost in *Pichia* and *Spathaspora* lineages. In family 4266, Cs186 is a putative result of many duplication events inside the *C. sojae* lineage (Fig. 1d). The high duplication rate coupled with high conservation of these genes suggests that these transporters are dosage-dependent as reported for hexose transporters [38]. For dose-dependent genes, it is potentially adaptive to increase gene number maintaining sequences features because it implies in higher transcription and a larger number of proteins working in the cell. In this way, transporter genes that undergo recent duplication events might be evolving under a selective process. By using an evolutionary approach for prospecting xylose transporters candidates for industrial applications, we hypothesize that transporters from our dataset more closely related to xylose transporters known from literature would have a similar capacity on uptaking this pentose. We then selected closely grouped genes to these transporters as candidates, such as Cs4130 and Cs3894 (close to *GXF1*), as well as some more distantly grouped—Cs186, a whole orthogroup distant from *XUT1*, *XUT3* and XylhpI; and Cs2608, close to *GXS1*. Based on sequence, Cs186, Cs3894 and Cs4130 are facilitators and Cs2608 is a symporter.

### *C. sojae* transporters mediate uptake of different carbon sources

**Fig. 2:**
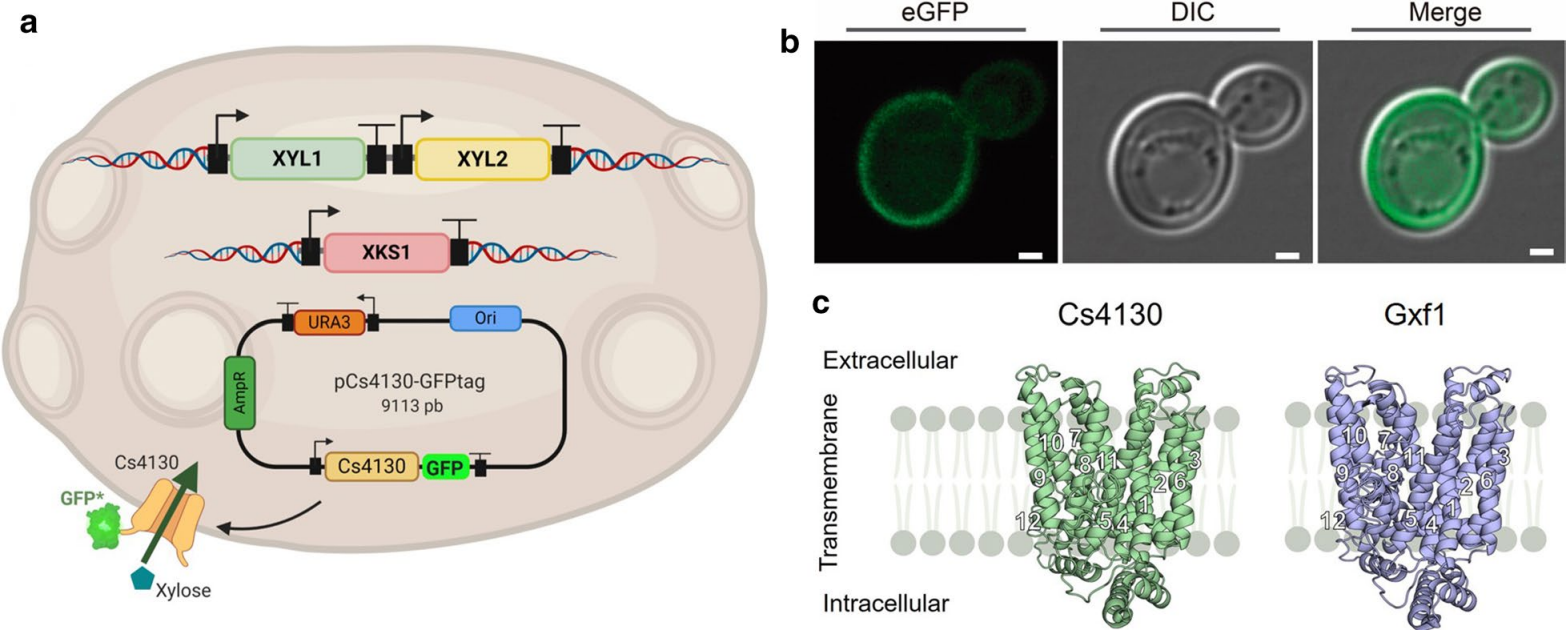
Subcellular localization of tested transporters in *S. cerevisiae* cells. **a** Design of EBY_Xyl1 cells with an integrated XR/XDH/XKS pathway and a multi-copy vector expressing the transporter fused with a codon-optimized eGFP-ymUkG1 (Created with BioRender). **b** A confocal image confirms the presence of a fluorescence halo in the cell periphery indicating the correct localization of the transporter in the cell membrane. DIC and fluorescence images were taken by a confocal microscope and merged. Scale bars = 1 μm. **c** Crystallographic and modeled structures of Cs4130 and Gxf1, respectively, embedded in the cytoplasmatic membrane. Cs4130 and Gxf1 show the typical fold of MFS transporters. The 12 transmembrane α-helix in Cs4130 and Gxf1 were assigned based in XylE

The substrate spectrum from the selected sugar transporters Cs186, Cs2608, Cs3894, and Cs4130 was evaluated in the strain EBY_Xyl1, a modified yeast cell derived from EBY.VW4000, which lacks transporters of the families *HXT1*-*17* and *GAL2*, and is unable to grow on glucose as the sole carbon source [39]. EBY_Xyl1 was constructed with the integration of an expression cassette containing the genes *XYL1* and *XYL2* from *S. stipitis* and an additional copy of xylulokinase (*XKS1*). The three genes were expressed under the control of different strong constitutive promoters of genes from the glycolytic pathway of *S. cerevisiae* (Additional file 4: Table S2). The candidate transporters were cloned in a multi-copy vector pRS426 using the promoter and terminator regions of *TDH1* from *S. cerevisiae*. As a positive transport-system control, the traditional xylose-facilitator Gxf1 from *C. intermedia* was chosen, since this transporter is well-known as the best heterologous xylose transporter already described in the literature [21, 33]. The plasmids expressing the cloned putative C5 transporters were used to transform the EBY_Xyl1 yeast cell. The candidate transporters were fused to a codon-optimized GFP-mUkG1 sequence to confirm the correct cellular localization in the yeast cell membrane and observed by confocal microscopy. Yeast cells expressing the GFP-tagged plasmids presented a fluorescence halo in the cell periphery indicating the correct localization of the transporters in the cell membrane (Fig. 2). However, the presence of fluorescence in the cytoplasm in a few yeast cells was observed, indicating possible degradation through a regulatory post-translational modification [40] or an unfolded protein response (UPR) triggered by overexpression of the heterologous transporter protein [41].

**Fig. 3:**
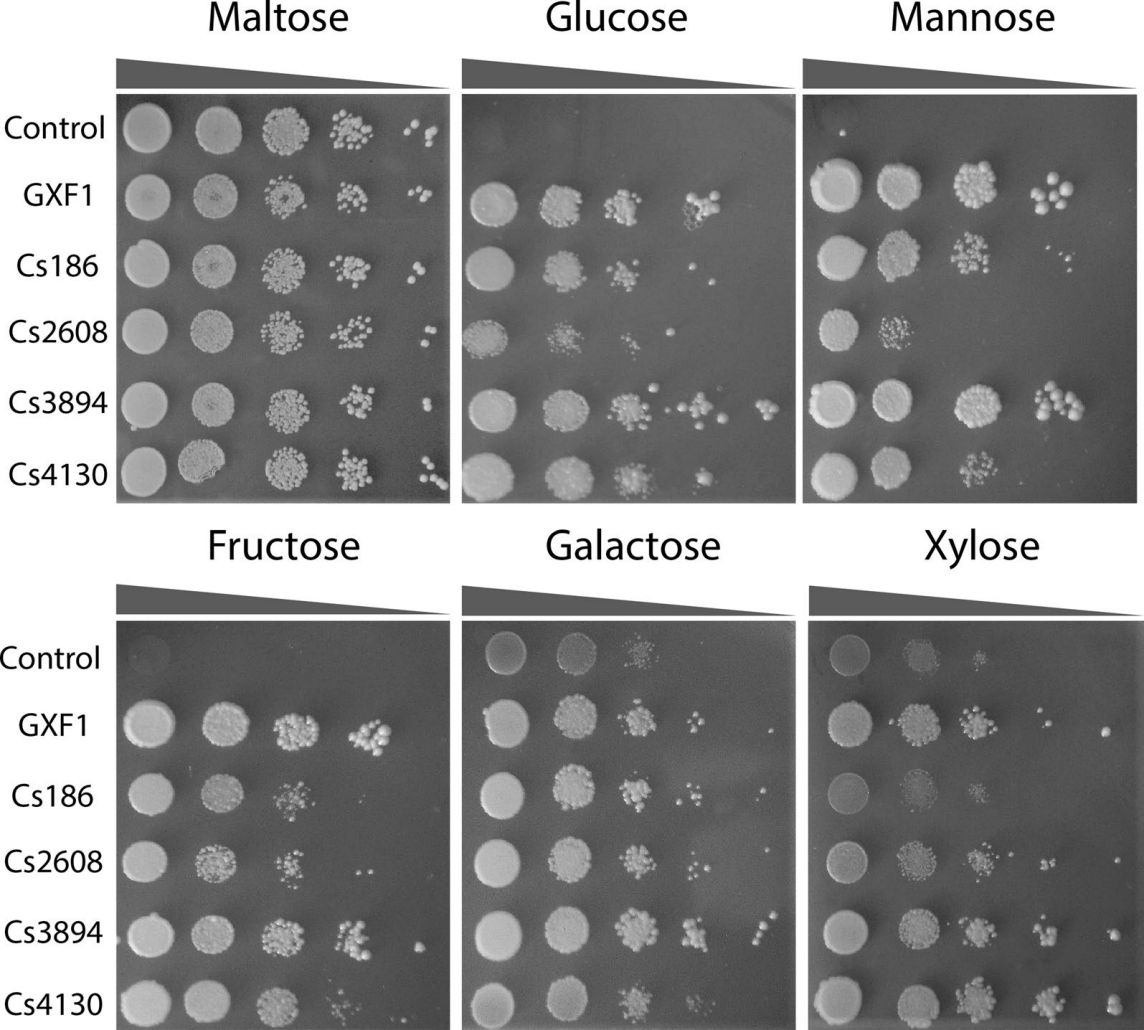
Functional profile of sugar affinities of *C. sojae* transporters. Drop-assay analysis on different sugars, 20 g/L, of EBY_Xyl1 strain expressing the indicated transporters and the control vector pRS426. The cultures were incubated at 30 °C for 3 days (maltose), 5 days (glucose, mannose, fructose and galactose) or 7 days (xylose). Yeast cells inocula were grown in YNB complete medium without uracil supplemented with maltose for 24 h. Initial OD_600_ was established at 1 before a serial dilution. All experiments were performed in triplicate

We analyzed the substrate spectrum of EBY_Xyl1 mutants carrying the specified sugar transporters using six different carbon sources on solid culture medium – 2% maltose (control), mannose, fructose, xylose, glucose, and galactose. The transformants were grown overnight to the exponential phase and spotted in tenfold serial dilutions onto solid culture medium. This analysis showed that *C. sojae* transporters restored the growth of EBY_Xyl1 strain in all sugars evaluated (Fig. 3). Curiously, the control strain EBY_Xyl1 carrying pRS426 plasmid showed a slight background on galactose and xylose. Parental strain EBY.VW4000 was previously described to show basal growth on galactose [39]. As this background was similar comparing these two sugars, the remaining transporters on the EBY.VW4000 strain which confers growth on galactose probably could be responsible for this behavior observed for xylose. However, for the other sugars, except maltose (control), no growth was demonstrated using the control EBY_Xyl1_pRS426. All transporters were functional and able to restore growth in all evaluated carbon sources showing a broad substrate affinity. The only exception was Cs186, which presented a slight background growth similar to pRS426 control on xylose, indicating that this transporter is unable to assimilate this sugar. Cs3894 and Gxf1 were able to support better and similar patterns of growth on glucose, mannose and fructose compared to the other transporters. The strain expressing the Cs2608 presented a poor growth on mannose and glucose compared to the other substrates. Cs3894 and Cs4130 presented a superior growth on 20 g/L of xylose and, interestingly and not expected, the growth of the strain expressing Cs4130 was better on xylose than glucose.

### Growth at different ranges of xylose concentrations

**Fig. 4:**
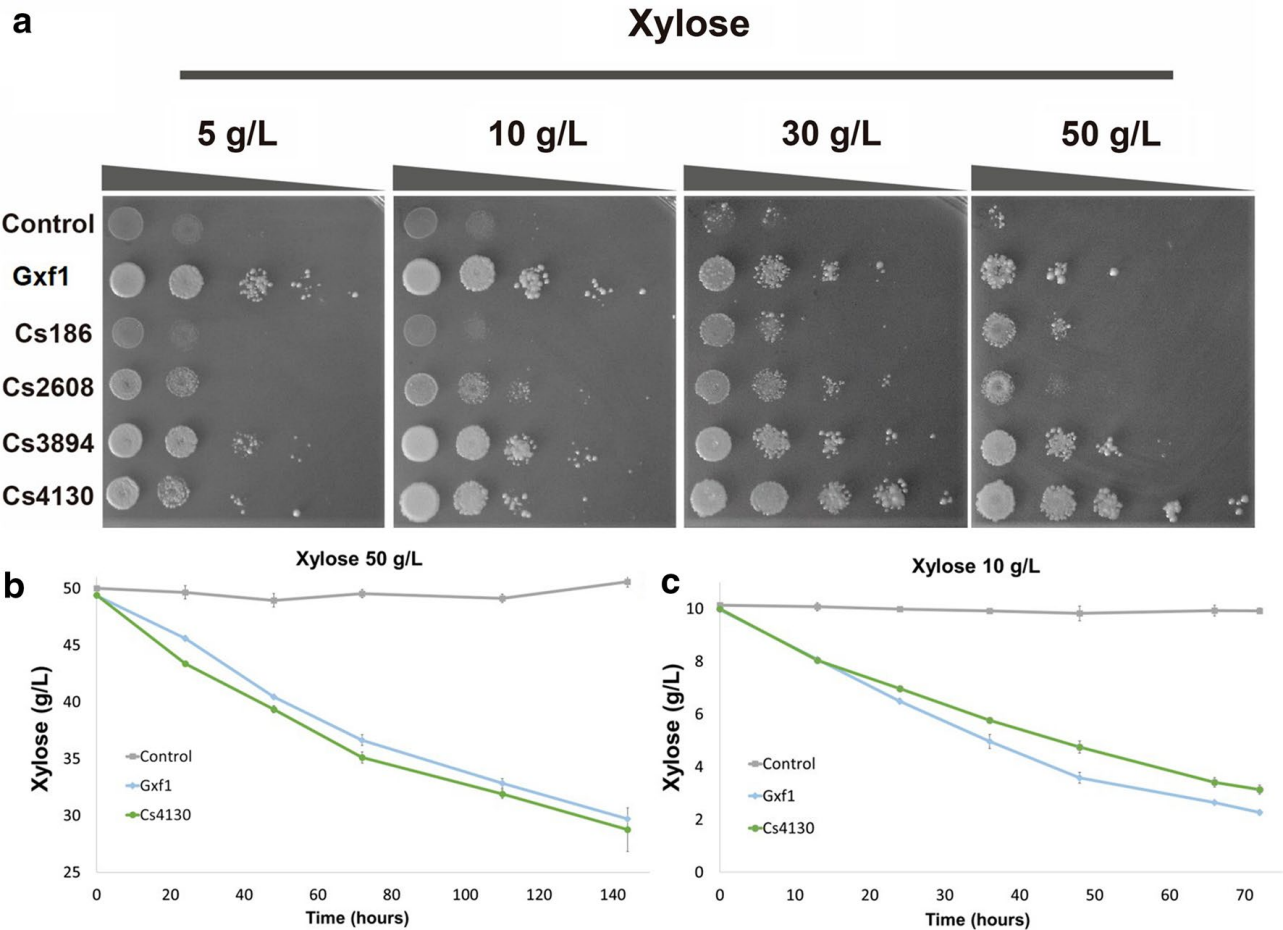
Functional profile of concentration-dependence of *C. sojae* transporters. **a** Growth on different range of xylose concentrations showing an uptake/concentration dependency. All experiments were performed in triplicate. High-cell density fermentation of Cs4130 and Gxf1 in xylose at 50 g/L (**b**) and 10 g/L (**c**). The fermentations were carried out on YNBX medium at 30 °C, 200 rpm. Control represents the EBY_Xyl1 harboring empty vector pRS426. Error bars represent standard deviation from average measures from triplicates

The growth of EBY_Xyl1 mutants carrying *C. sojae* transporter genes and *GXF1* as positive control were compared in solid medium with 0.5, 1, 2, 3 and 5% of xylose as the sole carbon source (Fig. 4a). The facilitator Gxf1 restored growth in all xylose concentrations evaluated. Analyzing the results of Gxf1, it is possible to notice a decrease in growth with increasing concentrations of xylose. Although this facilitator allows a higher growth of EBY_Xyl1, compared to all other *C. sojae* transporters on low xylose concentrations, 5 and 10 g/L, on intermediary and high concentrations, inhibition was observed. Surprisingly, the *C. sojae* transporters Cs3894 and Cs4130 demonstrated an opposite behavior allowing improvements on growth in intermediary and higher xylose concentrations, 30 g/L and 50 g/L, respectively (Fig. 4a). The EBY_Xyl1 cell expressing Cs4130 showed a better growth pattern compared to the other transporters in high concentrations of xylose, showing a natural loss of inhibition in contrast to commonly observed in Gxf1. The spot-assay experiment in different xylose concentrations were repeated three times, and each experiment was conducted in triplicate to confirm the growth pattern.

The spot-assay results were also confirmed in liquid media in a high-cell density fermentation assay using EBY_Xyl1_pRS426 as a control strain and xylose as the only source of carbon. Since Cs4130 presented the most interesting results, we focused further experiments in this facilitator. Yeast cells expressing Cs4130 were able to assimilate xylose and grow in both 10 and 50 g/L, although a higher xylose consumption rate was observed in superior concentrations (Fig. 4b, c). For both concentrations, in the first hours of analysis, productivity was higher, but decreased during fermentation, probably due to redox balance limitations from the XR–XDH pathway. As expected, cells expressing Gxf1 maintained the same consumption profile observed in the spot-assay. Comparing both transporters on 50 g/L, Cs4130 allowed faster assimilation with an increase of 30% in xylose consumption rate compared to Gxf1 in 24 h. However, on lower xylose concentrations, Gxf1 allowed a faster growth and improved xylose consumption rate by 49,5% in 24 h (Fig. 4b, c).

### Simultaneous consumption of glucose and xylose on Cs4130

**Fig. 5:**
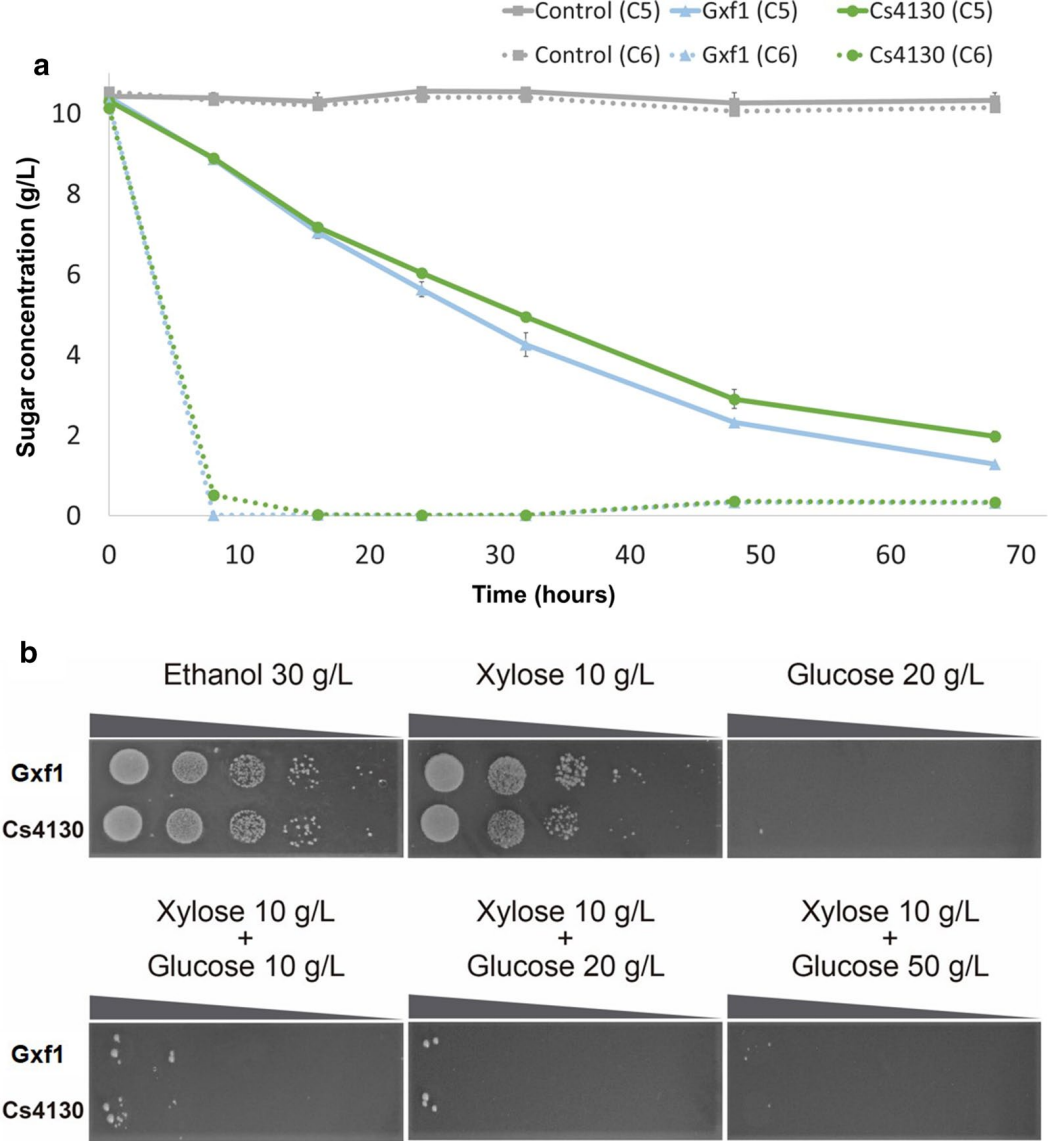
Glucose inhibition of Cs4130 in glucose and xylose mixtures. **a** High-cell density fermentation of Cs4130 and Gxf1 in xylose/glucose co-fermentation. Xylose and glucose consumption are represented by full and dotted lines, respectively. Fermentations were conducted on complete medium with 10 g/L of xylose and 10 g/L of glucose at 30 °C, 200 rpm. Control represents the EBY_Xyl1 harboring empty vector pRS426. Error bars represent standard deviation from average measures from triplicates. **b** Drop-assay analysis of EBY_Xyl1_*hxk*^*0*^ strain expressing the indicated transporters. Cells were grown on complete medium without uracil supplemented with 3% ethanol for 56 h before a serial dilution. The initial OD_600_ was established on 1. Plates were incubated at 30 °C for 10 days

Simultaneous consumption of glucose and xylose in a high-cell density fermentation using a C6/C5 mixture was also evaluated, with 10 g/L of each sugar and EBY_Xyl1 strain expressing Cs4130 and Gxf1 (Fig. 5a). This fermentation showed a faster glucose consumption in the first 10 h. A very small amount of xylose was consumed in this period, confirming, as expected, a higher affinity for glucose compared to xylose. As an example, the facilitator Gxf1 is known to have a higher glucose affinity (*K*_m glucose_ = 2.0 ± 0.6) compared to xylose (*K*_m xylose_ = 48.7 ± 6.5), [34]. The preferential glucose consumption confirmed that Cs4130 does not bypass glucose inhibition, similarly as observed for Gxf1. As the concentrations used were low (10 g/L), cells expressing Gxf1 consumed the sugars faster.

To further evaluate the glucose inhibition of Cs4130, from EBY_Xyl1, the strain EBY_Xyl1_*hxk*^*0*^ was constructed by disrupting the glycolytic pathway through gene knockout of three hexokinases: *HXK1*, *HXK2* and *GLK1*, and further restored sugar uptake by expression of individual heterologous transporters, Cs4130 and *GXF1*. The derived strain assimilated glucose from extracellular media but was not capable to metabolize this sugar as a carbon source (Fig. 5). Therefore, it is a platform to analyze glucose inhibition in a sugar mixture. Since glucose or maltose cannot be used for growth, we used 3% ethanol as a positive control. EBY_Xyl1_*hxk*^*0*^ strains harboring individual heterologous transporters, showed, as expected, growth on xylose and impairment on glucose uptake. The glucose inhibition test was performed in three different concentrations of C5/C6 mixtures on solid media. The xylose concentration was maintained on 10 g/L and glucose was added to final concentrations of 10 g/L, 20 g/L and 50 g/L, respectively. The inhibition test showed that yeast cells expressing both transporters were unable to restore significant growth on the different mixtures, even on low glucose concentrations (Fig. 5b). This phenotype indicates that these heterologous transporters show a natural higher preference for glucose over xylose, which leads to growth impairments on *hxk*^*0*^ strains on the presence of both sugars.

### Docking and normal mode analysis (NMA) point out different behavior of xylose-bound Cs4130 and Gxf1

The modeled structures of Cs4130 and Gxf1 were acquired in the I-TASSER server [42], both showing the typical fold of MFS transporters: 12 transmembrane (TM) α-helix with the N- (TM1–TM6) and C- (TM7–12) termini facing the intracellular side [14] (Fig. 2c). High TM-scores (> 0.6) were recorded for GLUT transporters, typical of mammals (PDB codes: 5C65, 4YBQ and 4PYP); XylE and GlcP_SE_, from bacteria (4GBY, 4LDS); and STP10, from plant (6H7D) for both Cs4130 and Gxf1 models. XylE, a d-xylose: H^+^ symporter found in *Escherichia coli* shows 27.02 and 25.56% identity (amino acids) with Cs4130 and Gxf1, respectively. XylE was adopted as a facilitator model throughout this work given the availability of its outward-facing/partly occluded crystallographic structure bound to xylose (4GBY) as well as the distinction of some residues essential for sugar transport [43], which was extended to Cs4130 and Gxf1 in this work.

**Fig. 6:**
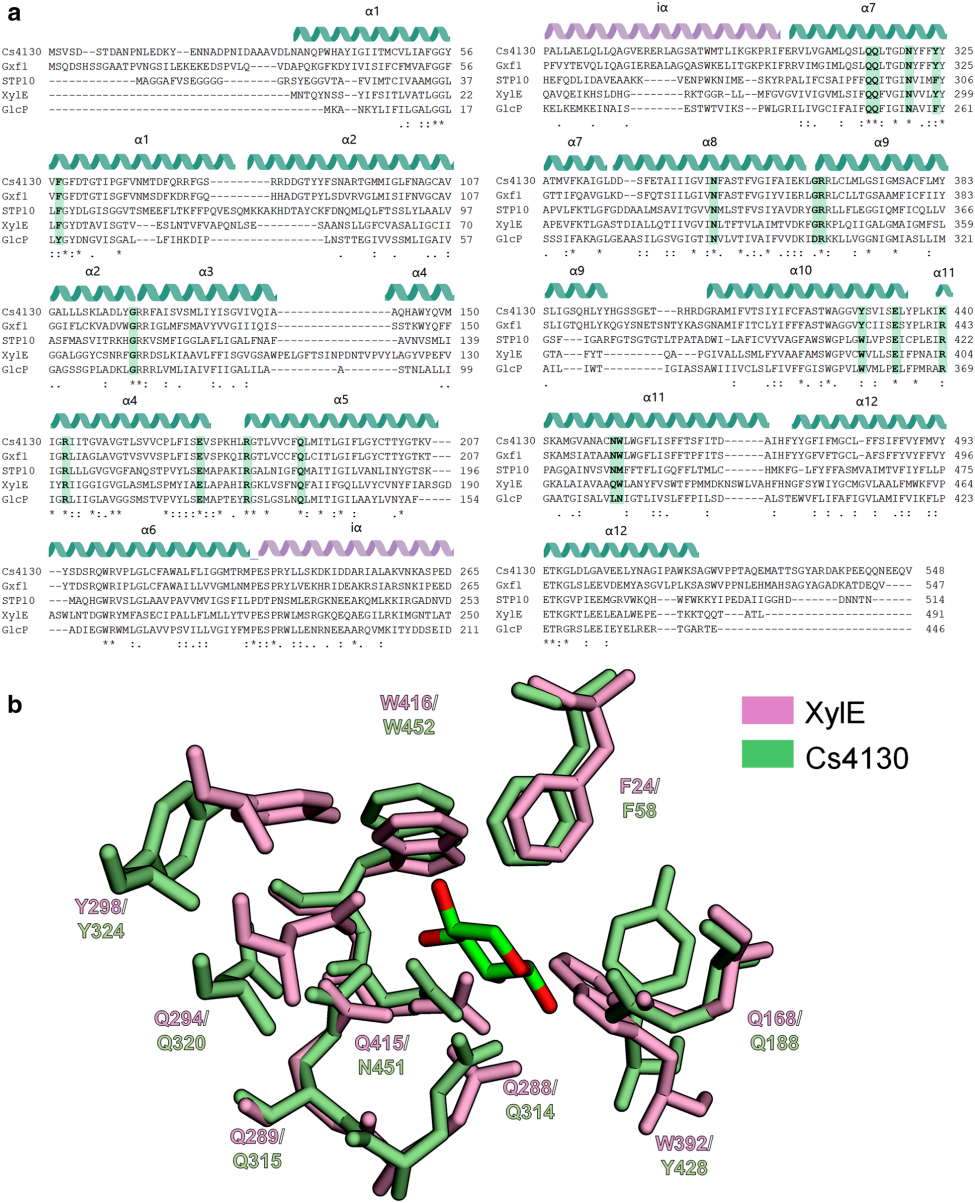
Alignment of amino acid sequences of Cs4130, Gxf1, STP10, XylE and GlcPSE (**a**) and superposition of XylE (pale pink) and Cs4130 (pale green) intermembrane residues related to xylose (green) transport (**b**). The residues mutated in XylE and their counterparts in Cs4130, Gxf1, STP10, and GlcPSE are highlighted in green. The position of α-helix was signed based in XylE. α, α-helix; iα, intracellular α-helix

The docking analysis of Cs4130 and Gxf1 with glucose, fructose, galactose, mannose and xylose showed that all these sugars can bind to both transporters (Table 1). Cs4130, Gxf1 and XylE show higher binding affinities (lower binding energies [kcal/mol]) to glucose than xylose. Regarding xylose, a slightly low binding energy was recorded for Cs4130 compared to Gxf1 and XylE (Table 1). Mutation of specific residues in XylE leads to a decrease or abrogation of xylose transport [43]. Their counterparts in Cs4130, Gxf1, STP10 (from *A. thaliana*) and GlcP_SE_ (from *Staphylococcus epidermidis*) are strongly conserved suggesting their importance for sugar transport (Fig. 6, Additional file 5: Table S3).

**Table 1:** Binding energies (kcal/mol) of Cs4130, Gxf1 and XylE with monosaccharides through molecular docking analysis

GRID SCORE (kcal/mol)					
Transporter monosaccharides					
	Glucose	Fructose	Galactose	Mannose	Xylose
Cs4130	− 32.9	− 29.7	− 30.5	− 31.6	− 28.8
Gxf1	− 29.3	− 29.9	− 29.3	− 29.8	− 26.0
XylE	− 31.0	− 30.4	− 33.3	− 33.9	− 27.4

**Fig. 7:**
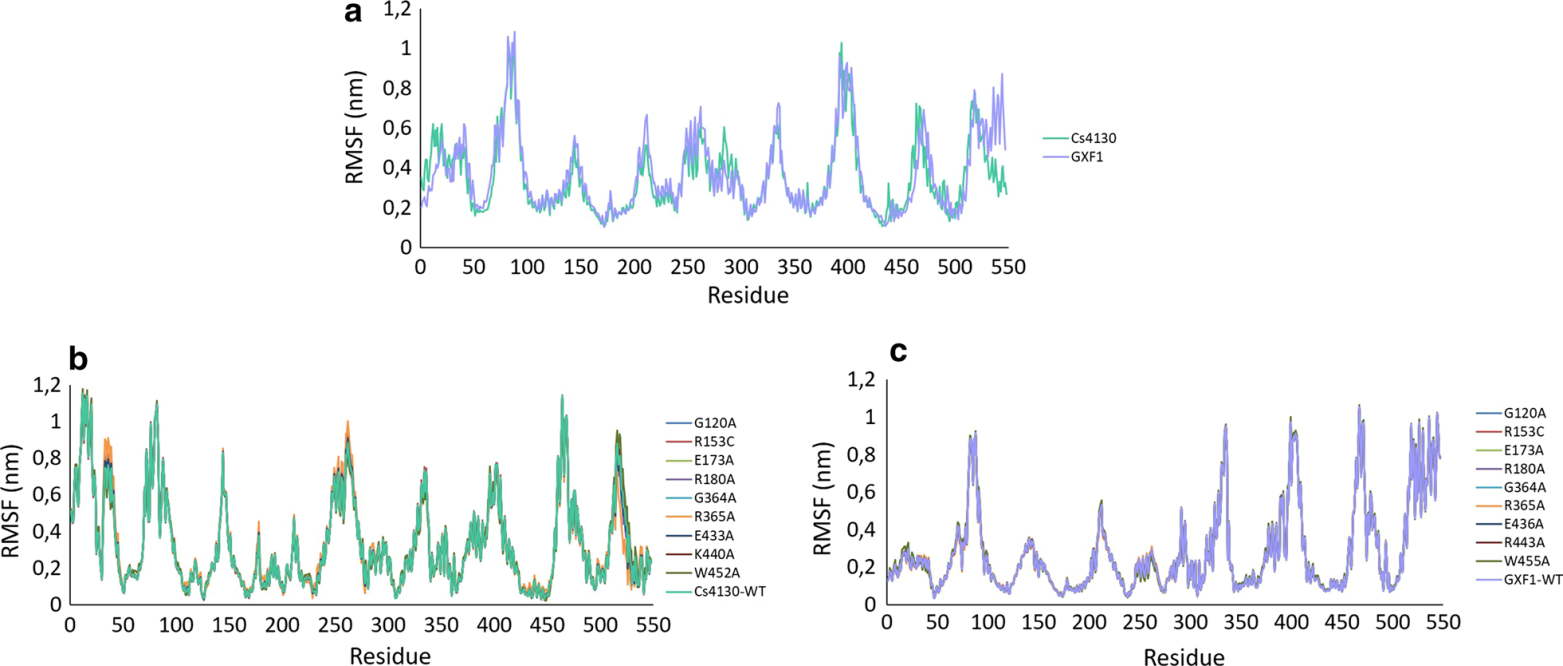
Evaluation of the dynamical regime through non-linear normal mode analysis. Comparison between WTs Cs4130 and Gxf1 (**a**). The low-frequency mode 6 for Cs4130 and Gxf1 (with respective mutants) are shown in **b** and **c**, respectively. The displacement of the residues was evaluated using the root mean square fluctuation (RMSF)

An extensive comparison of XylE with Cs4130 and Gxf1 was made to infer the molecular mechanisms related to the improved xylose assimilation by Cs4130 over Gxf1 at higher concentrations. The distance to xylose (DtX, Å) of all residues mutated in XylE [43] was measured using 4GBY as reference (Table 2) to understand how the residues interact with xylose. Given the conservation/superposition of the residues mutated in XylE in both Cs4130 and Gxf1 (Fig. 7, Additional file 5: Table S3), the distance to the ligand was considered as the same for all targets (Table 2). Thus, the xylose binding was evaluated by docking (residues close to xylose) or normal mode analysis (residues far from xylose). It is noteworthy that small variations in the binding energy (around 0.6 kcal/mol) of wild-type (WT) XylE compared to the variants Q168A, Q288A, N289A, N294A, Y298A, W392A, Q415A and W416A (near to xylose, < 5 Å) may be enough to abrogate the xylose transport as seen in Sun et al. [43]. This conclusion is corroborated by the molecular docking analysis of N325A. The binding energy of the N325A is equal to the WT (− 27.4 kcal/mol) supporting the molecular docking procedure by agreeing with the functional studies [43] (Table 2). When Q188 (Q168 in XylE), Q314 (Q288 in XylE), Q315 (Q289 in XylE), N320 (Y298 in XylE), Y324 (Y298 in XylE) and Y428/Y431 (W392 in XylE), N451/N454 (Q415 in XylE) and W452/W455 (W416 in XylE) in Cs4130 and Gxf1 were mutated to alanine and evaluated through molecular docking analysis, the Cs4130 mutants Q188A (variation in the binding energy compared to WT Cs4130: 2.4 kcal/mol), Q314A (0.7 kcal/mol), N320A (0.6 kcal/mol), Y324A (0.7 kcal/mol) and Y428A (0.9 kcal/mol) and N451A (0.8 kcal/mol) would probably result in altered xylose transport compared to WT Cs4130 since variations in the binding energy of around 0.6 kcal/mol is sufficient to abrogate xylose transport in XylE. For Gxf1, mutants Q314A (variation in the binding energy compared to WT Gxf1: 1.1 kcal/mol), N320A (0.8 kcal/mol), Y431A (0.7 kcal/mol), N454A (0.6 kcal/mol) and W455A (0.6 kcal/mol) would have the xylose transport affected (Table 2). At this stage, it is not possible to predict if the mutations could benefit or not the xylose transport in both Cs4130 and Gxf1.

**Table 2:** Distance between the residue mutated in XylE to xylose (DtX) in Angstrom (Å) and the binding energies (kcal/mol) of Cs4130, Gxf1, XylE and variants complexed to xylose through molecular docking analysis

	XylE	Cs4130	Gxf1	DtX (Å)
	Residue (kcal/mol)			
Wild-type	− 27.4	− 28.8	− 26.0	**–**
Mut1	F24A (− 27.6/0.2)	F58A (− 27.8/1.0)	F58A (24.9/1.1)	*3.9*
	G83A	G120A	G120A	22.0
	R133C	R153C	R153C	11.7
	E153A	E173A	E173A	17.8
	R160A	R180A	R180A	14.3
Mut 2	Q168A (− 28.3/0.9)	Q188A (− 26.4/2.4)	Q188A (− 25.5/0.5)	*2.3*
Mut 3	Q288A (− 26.4/1.0)	Q314A (− 28.1/0.7)	Q314A (− 24.9/1.1)	*2.9*
Mut 4	Q289A (− 26.6/0.8)	Q315A (− 28.5/0.3)	Q315A (− 26.1/0.1)	*3.0*
Mut 5	N294A (− 28.1/0.7)	N320A (− 28.2/0.6)	N320A (− 25.2/0.8)	*2.9*
Mut 6	Y298A (− 26.4/1.0)	Y324A (− 28.1/0.7)	Y324A (− 25.8/0.2)	*4.3*
Mut 7	N325A (− 27.4/0)	N349A (− 26.9/1.9)	N349A (− 25.7/0.3)	6.7
	G340A	G364A	G364A	15.8
	R341A	R365A	R365A	14.3
Mut 8	W392A (− 26.6/0.8)	Y428A (− 27.9/0.9)	Y431A (− 25.6/0.7)	*3.1*
	E397A	E433A	E436A	14.2
	R404A	K440A	R443A	16.1
Mut 9	Q415A (− 26.8/0.6)	N451A (− 28.0/0.8)	N454A (− 26.6/0.6)	2.7
Mut 10	W416A (26.5/0.9)	W452A (− 28.5/0.3)	W455A (− 25.4/0.6)	*4.8*

Residues close and far from ligand (xylose) are highlighted in italics and underlined, respectively

The energy of binding (kcal/mol) of mutants and the difference from the wild-type are between parentheses

*Mut* mutant

Other residues, beyond the ones mutated in XylE, were chosen in Cs4130 and Gxf1 based in their closeness to xylose (< 5 Å) to be evaluated by docking analysis. Changes in xylose transport are expected in mutants I191A (variation in the binding energy compared to WT Cs4130: 1.4 kcal/mol), I195A (1.9 kcal/mol) and F419A (1.5 kcal/mol) for Cs4130, but only in mutant I195A (1.2 kcal/mol) and Y431A (0.7 kcal/mol) regarding Gxf1 (Additional file 6: Table S4). The differences in the binding energy of variants of Cs4130/Gxf1 compared to their respective WTs (Table 2 and Additional file 6: Table S4) suggests that altered xylose transport is more likely to happen in Cs4130 than in Gxf1.

The dynamical regime of the WTs Cs4130/Gxf1 and variants (mutations in residues far from xylose) were evaluated using the non-linear Normal Mode Analysis (NOLB software) [44] through the root-mean square fluctuation (RMSF). The RMSF was calculated as the average of the 10 lowest vibrational modes and an average of all 20 frames per mode. The WTs Cs4130 and Gxf1, despite the structural similarity (RMSD = 1.14 Å), show a different fluctuation pattern mainly in the N and C-terminal ends and residues 31, 41–49, 67, 71, 82–89, 145,146, 208–216, 253–258, 281–290, 335, 394–412, 438, 459–493 and 509–515 (Fig. 7a), making the comparison between mutants of transporters not so informative as by molecular docking analysis. Consequently, the influence of residue mutations must be compared only with its own WT. The residues G120, R153, E173, R180, G364, R365, E433 (E436 in Gxf1) and K440 (K443 in Gxf1) with distances from xylose underlined in Table 2, were mutated in Cs4130 and Gxf1 and their dynamical regimes were compared with the respective WTs. Figure 7b, c shows the RMSF average of Cs4130 and Gxf1, respectively. The mutation R365A in Cs4130 changes the dynamical behavior of Cs4130 WT, what is mostly evident in the low-frequency modes 6–8 (Fig. 7b shows only the mode 6), mainly in the residues 32–41 (N-terminal), 257–263 (cytoplasmatic region) and 515–529 (C-terminal), probably resulting in altered xylose transport (Fig. 7b, Additional file 7: Figure S3, Additional file 8: Figure S4). For the other Cs4130 variants, the dynamical regimes were quite stable considering all low-frequency modes as also observed for Gxf1 (Fig. 7c).

## Discussion

Metabolic engineering of microbial platforms for 2G biorefineries relies on the development of efficient transport systems to capture all the sugars from biomass [10, 12]. Xylose uptake across the plasma membrane is a critical limiting step in recombinant yeast strain development, reducing the efficiency of downstream pathways and conversion in high value-added bioproducts. Over the course of evolution, *S. cerevisiae* was highly equipped with a diverse and multipurpose transport system in order to deal with different conditions and challenging environments. The whole-genome duplication event expanded the repertoire of glucose transporters that *S. cerevisiae* has at its disposal and allows efficient growth in a wide range of glucose concentrations [45]. *S. cerevisiae* has 18 genes encoding hexose transporters—Hxt1p–Hxt17p and Gal2p, which belong to MFS. The endogenous transporters differ regarding the degree of glucose affinity, from low (*K*_m_ of 50–100 mmol/L) to high-affinity (*K*_m_ of 1–2 mmol/L), and are dependent on glucose concentration in the extracellular environment [36, 46]. The transport system is also fine regulated at the transcriptional and post-translational levels to adapt to different glucose concentrations [47]. As xylose fermentation was probably not a strong selective pressure in *S. cerevisiae* evolution, engineered strains of *S. cerevisiae* do not harbor all those transport system options or a regulated signaling system that allows pentose uptake in a range of different concentrations. Using an *in vivo* biosensor panel associated with three major signaling pathways, a recent work showed that an engineered C5 *S. cerevisiae* strain senses xylose differently from glucose. The signal triggered at high concentrations of xylose was similar to the carbon starvation signal observed at low glucose levels [48, 49]. In addition to the differences observed in cell signaling, in order to overcome transport limitations that result in low assimilation rates, a global modification at the xylose transport system is necessary with the integration of facilitators capable of transporting xylose in a wide range of sugar concentrations.

Natural evolution already resulted in many pentose-assimilating yeast species which transport xylose with high and low affinities and the functional expression of only a few heterologous transporters were demonstrated to improve xylose uptake in recombinant *S. cerevisiae* strains [21, 50–53]. For example, the xylose transporter Sut1 isolated from *S. stipitis* was able to improve xylose consumption by 25% and increase ethanol yield by 17% on recombinant *S. cerevisiae* cells [50]. The transporters Xyp29, AXT1, XYLH, and MGT05196 from C5 yeasts *S. stipitis* [52], *Pichia guilliermondii* [54], *D. hansenii* [55] and *Meyerozyma guilliermondii* [56], respectively, were also able to restore significant xylose growth on expressing strains. Even *A. thaliana* xylose transporters, At5g17010 and At5g59250, were responsible for 25% and 40% of improvements in xylose consumption rate, respectively, on *S. cerevisiae* strains [57]. As highlighted above, the facilitator Gxf1 isolated from *C. intermedia* [34] has been described as the best heterologous xylose transporter, although it improved growth in xylose only at low sugar concentrations (4 g/L), and no improvement was observed for concentrations higher than 15 g/L [21, 26, 33]. Gxf1 shows an intermediate affinity to xylose and a glucose preference 20-fold higher [34].

By assessing microbiomes such as the digestive tract of plague insects and decayed biomasses, we isolated pentose yeast species capable of naturally using xylose (Additional file 2: Table S1). The cultivation in xylose by successive batches limited the naturally diverse population by substrate competition and allowed the isolation and molecular identification of only a few species able to consume xylose efficiently. Since these strains compete to grow in xylose, an efficient transport system to uptake the C5 sugar was expected. The yeast *C. sojae* was selected after comparative fermentations as a potential source of high-affinity xylose transporters. *C. sojae* was isolated from the intestine of a *Coleoptera* larvae collected internally from an energy cane stalk (Additional file 2: Table S1). From genome analysis [58], well-known eukaryotic xylose transporters, such as *HXT7* [20, 22, 59], *GAL2* [20, 22], *XUT1* [21], *XUT3* [21], *SUT1* [50, 51], *GXS1* [28, 29, 34], *GXF1* [25–27, 34], *Xylhp* [21] and other members from MFS were used as baits to select the transporter-encoding genes Cs186, Cs2608, Cs3894 and Cs4130. From phylogenetic comparative analysis (Fig. 1) two of the elect *C. sojae* transporters, Cs4130 and Cs3894 showed similarity to uniport transporters such as *GXF1*, *HXT7* and *GAL2*, whereas Cs186 was related to *XUT3* from *S. stipitis*. However, Cs2608 was more related to symporter transporters, such as *GXS1*. Therefore, these sequence similarities suggest that Cs186, Cs3894 and Cs4130 transporters are novel members of uniport transporters and Cs2608 represents a new xylose/glucose–H^+^ symporter.

All selected *C. sojae* transporters were evaluated on EBY_Xyl1 strain, a modified version of EBY.VW4000 [39], expressing the XR/XDH/XKS xylose consumption pathway (Additional file 4: Table S2). *C. sojae* transporters were able to restore growth of EBY_Xyl1 in all sugars evaluated, showing a broader substrate specificity (Fig. 3). Regarding Cs186, its low xylose transport capacity may be explained not only by the distant relation to known xylose carriers but also by the presence of many identical copies on *C. sojae* genome (Cs186, Cs173, Cs183, Cs136) as shown in the polytomy in Fig. 2d. As transporters have a dose-dependent effect, this low capacity could be compensated by the extra copies, which together supply xylose uptake for *C. sojae* cells. Cs2608 is a symporter and only a few examples were functionally expressed on *S. cerevisiae* previously in the literature. Despite being functional and restoring EBY_Xyl1 growth, Cs2608 performed poorly on most of the tested sugars, especially glucose and mannose (Fig. 3).

The growth profile in xylose was different comparing *C. sojae* transporters and Gxf1 in crescent xylose concentrations (Fig. 4a). While the growth of Gxf1 strain decrease as the xylose concentration increase, strains expressing Cs3894 and Cs4130 showed superior xylose uptake at concentrations up to 50 g/L (Figure 4a). Depending on the type of process used to release sugars from biomass, xylose concentrations in 2G hydrolysates can reach high values, with concentrations of 65 g/L [60], 85 g/L [61], and even higher values [62] already reported in the literature. Therefore, Cs3894 and Cs4130 are profitable candidates for xylose uptake due to their natural loss of inhibition at higher concentrations. Interestingly, Cs4130 restores the growth of recombinant strains even at high xylose concentrations, presenting an opposite behavior to Gxf1. Since Cs4130 enabled better growth compared to Cs3894, especially in higher concentrations of xylose, further experiments were done with only the first facilitator. The fermentation in liquid media confirmed the pattern of growth of Cs4130 observed in spot-assay experiments, presenting higher productivity in 50 g/L against 10 g/L of xylose. A more subtle difference between Cs4130 and Gxf1 was observed in fermentation in the liquid medium compared to growth in a solid medium, since transport differences may have been attenuated by limitations on the downstream pathway. EBY_Xyl1 cells expressing Cs4130 improved xylose consumption rates at 50 g/L when compared to Gxf1 during liquid fermentation, while the opposite was observed at 10 g/L, maintaining the same consumption profile observed in the spot-assay experiment (Fig. 4b, c). The design of microbial cell factories to efficiently convert biomass sugars requires the introduction of transporters capable to assimilate xylose in different concentrations available in lignocellulosic hydrolysates. In this context, the combination of Gxf1 and Cs4130 to engineer a xylose-fermenting *S. cerevisiae* strain can be an interesting approach, assuring efficient xylose utilization in 2G hydrolysates.

Besides differences in transporter affinities, substrate-induced ubiquitination could also explain the differences observed between both transporters. Membrane transporter protein can be removed from the cytoplasmic membrane and degraded in the absence of glucose [63, 64]. For example, Hxt1 and Hxt3 are low-affinity transporters, expressed in the presence of a high concentration of glucose and degraded in the absence of glucose [47, 65, 66]. However, the molecular mechanisms that could explain if different concentrations of xylose trigger endocytosis are not clear and further investigation is required.

Simultaneous uptake of glucose and xylose is highly desirable for engineered 2G strains. Cells expressing a heterologous xylose assimilation pathway preferentially consume glucose and a diauxic growth is observed in hydrolysates. Glucose repression is caused mainly by competition for uptake through hexose transporters [67]. In order to investigate if the transport mechanism of Cs4130 alleviates the glucose inhibitory effect, we tested different growth conditions with mixtures of glucose and xylose. High-cell density fermentation with an equal concentration of both sugars (Fig. 5a), showed a preferential consumption of glucose in the first 10 h, indicating a higher preference for glucose over xylose. We disrupted glycolysis in EBY_Xyl1_*hxk*^*0*^, uncoupling glucose uptake from its metabolization, similarly as the strategy described by Farwick, et al. [31]. The mutant cell is unable to metabolize glucose when hexokinase genes (*HXK1*, *HXK2* and *GLK1*) are knocked out [31, 68]. EBY_Xyl1_*hxk*^*0*^ expressing *GXF1* and Cs4130 showed strong glucose inhibition on a mixture of C5 and C6 (Fig. 5b). Higher glucose concentration on mixtures of C5 and C6 decreases the growth of the strain overall. A strong glucose inhibition was previously described for the native transporters Gal2p and Hxt7p using an *HXK* null strain. In the same C5/C6 mixture condition using 10 g/L of xylose and 50 g/L of glucose, a severe impairment in xylose uptake was observed for both transporters, showing a higher affinity for glucose over xylose [31].

Sugar transport in cells is a dynamic event where transmembrane proteins oscillate between the outward/occluded and inward/occluded states. Some residues make the path for the ligand mainly by short-range interactions, for example, hydrogen bonds, which are constantly redesigned to pull the sugar into the cytoplasmatic face [69]. The sugar-binding regions in XylE [43], Cs4130 and Gxf1 are conserved. The differences in the binding energy values seen in Table 1 emphasize the specificities of each transporter when the same monosaccharide is taken into consideration (except fructose). Probably, the non-identical residues in the vicinity of the residues bound to sugar promote local changes in the environment affecting the interactions.

A comparison of XylE experimental data [43] with the molecular docking analysis of this work suggests that the increase/decrease of binding energies of variants equal or higher than 0.6 kcal/mol compared to their respective WTs seems to be enough to affect xylose transport. The only exception is the residue F24A in XylE (F58A in Cs4130 and Gxf1), where a difference in binding energy equal to 0.2 kcal/mol affect xylose transport (Table 2). The TM1 harbors the residues F24 and F58 in XylE and Cs4130/Gxf1, respectively (Fig. 6a). The conserved motif G–G/F-XXX-G in TM1 is vastly found in facilitators which capture xylose [28]. XylE, Cs4130 and Gxf1 present this conserved motif where the “XXX” are represented by L22/L23/F24 Y56/V57/F58 and F56/V57/F58, respectively. Contrary to that observed for XylE, the mutation F40A (same position of F24 and F58 in XylE and Cs4130/Gxf1, respectively) in the transporter Gxs1 from *C. intermedia* increase the selectivity for xylose over glucose, being the best results attained for mutants F40M and F40S [28]. Also, mutation in the TM1 residue F79S (Y56 and F56 in Cs4130 and Gxf1, respectively) in Hxt7p favors the growth of *S. cerevisiae* in xylose as the sole carbon source and allows the co-consumption of xylose and glucose [70], revealing that a fine-tuning between the residues directly involved with the xylose transport and their vicinity should exist.

The potential contribution of residues distant to xylose for transport in Cs4130 and Gxf1 was evaluated by Normal Mode Analysis (NMA). NMA assumes that modes exhibiting the lowers frequencies describe the largest movements in a protein [71, 72]. The mutation R365A in Cs4130 changes the dynamical regimes mainly of the residues 32–41, 257–263 and 515–529 (Fig. 7b). A zoom view shows that residues adjacent to these ranges are also affected by R365A in a minor proportion (Additional file 7: Figure S3). Then, the variant R365A would probably result in a different transport phenotype for Cs4130, but not for Gxf1, which RMSF remains quite stable in all 10 low-frequency modes evaluated for all protein variants (Fig. 7c). It is important to emphasize that NMA uses simple constructions for the model, so small differences in the regime of fluctuations may be related to considerable variations in the dynamical behavior in physiological conditions. The residues affected by R365A are found mainly in the Cs4130 N and C-terminal regions. These portions, for both Cs4130 and Gxf1, do not fit with XylE crystallographic structure being promising sites to study the effect on sugar transport. Also, changes in the dynamical regimes of 257–263, intracellular face, could result in altered sugar transport given that loop regions are also important for the phenotype of transporters [36].

Some studies show that not only the residues directly involved in sugar binding are important for transport, but also those ones mapped far from sugar. Qureshi et al. [73] showed that polar interactions of residues about 15 Å from sugar are determinant for the transport of glucose and fructose in PfHT1 from *Plasmodium falciparum*. The authors concluded that the substrate promiscuity is not devoid to sugar-binding site, but substrate-gating dynamics. In the same way, N367 in Hxt36 (N325 equivalent in XylE and N349 in Cs4130 and Gxf1) was mutated by all amino acids and the best results concerning S*. cerevisiae* growth in glucose and xylose were achieved with small and non-polar residues (mainly alanine and glycine) [74]. In XylE, N325A also retained the capacity to transport xylose [43].

The transport of sugars through the cellular membrane is not an obvious event in nature. Several bonds are made and broken in a restrictive and insoluble micro-environment. From the functional and in silico data, it is hypothesized that Cs4130 is a more promiscuous transporter than Gxf1 given it supports higher concentrations of xylose. In addition, mutations in Cs4130 will probably result in a diversity of responses not observed in Gxf1, for example, placing Cs4130 as a model to obtain transporters with improved properties regarding the assimilation of substrates other than glucose.

## Conclusions

This work describes the novel xylose transporter Cs4130 and its functional expression in *S. cerevisiae* at high xylose concentrations, an important missing feature in C5 transporters previously described in the literature. The novel transporter was identified by accessing diverse microbiomes and using an evolutionary approach to prospect transporters candidates for industrial applications. The transport capacity of several targets was evaluated and Cs4130 stood out due to the natural loss of inhibition in high xylose concentrations. We also investigated the dynamic behavior of Cs4130 and conformational changes regarding xylose translocation, pointing out important residues related to transport in Cs4130 variants and revealing interesting aspects of xylose uptake mechanisms. These findings will be used for rational design procedures aiming for improved transport kinetics favoring the development of robust strains to face the challenges of 2nd generation industries.

## Methods

### Media and culture conditions

Yeast cells were grown on liquid YP medium (10 g/L yeast extract and 20 g/L peptone) supplemented with 20 g/L d-glucose (YPD) for cell propagation. For growth in xylose, d-glucose was replaced by 20 g/L d-xylose (YPX). For transformants selection, cells were grown at 30 °C in complete synthetic media YNB (6.7 g/L yeast nitrogen base without amino acids, Difco) supplemented with 1 g/L drop-out without uracil, 20 g/L glucose and 20 g/L agar [75]. YP was autoclaved at 121 °C for 20 min and YNB was filter-sterilized using 0.2-μm bottle-top filters. Strain EBY.VW4000, kindly provided by Prof. Eckhard Boles from Goethe university [39], and strain EBY_Xyl1 were grown in YNB with d-maltose instead of d-glucose, and EBY_Xyl1_ *hxk*^*0*^ were cultivated in 3% ethanol as carbon source. EBY_Xyl1 expressing transporter genes were grown in solid YNB supplemented with different carbon sources (d-galactose, d-mannose, d-fructose, d-xylose, d-glucose and d-maltose) for spot-assay analysis. High-cell density fermentation of EBY_Xyl1 expressing the heterologous transporters were performed on YNB supplemented with 5 g/L of casamino acids (Difco), 1 g/L of tryptophan (Sigma) and the correspondent carbon source, d-maltose, d-xylose or d-glucose. The yeast cells isolation media was adapted from Cadete et al. [76]: 6.7 g/L yeast nitrogen base without amino acids (Difco) supplemented with 1 g/L of complete drop-out, 7 g/L of d-xylose, 0.2 g/L of chloramphenicol. For solid media, 10 g/L d-xylose and 20 g/L agar were employed. The selection media for strains with pSH65 plasmid was supplemented with 300 μg/mL of zeocin (Thermo Scientific) on solid plates. For *URA3*/5-FOA counter-selection procedure, strains expressing pSH65 plasmid were grown in synthetic medium supplemented with 440 mg/mL of uracil and 1 g/L of 5-FOA (Zymo Research). For antibiotic resistance, 200 mg/L of geneticin and 200 mg/L of hygromycin was added to YPD for strains expressing the *KanMX4* (G418) [77] or *hph* [78] markers, respectively. Likewise, 300 μg/mL of zeocin was added for strains transformed with pSH65 [79]. Permanent stocks of yeasts and *E. coli* cells were mixed with glycerol before storage at − 80 °C.

### Isolation and screening of d-xylose-consuming yeasts

The C5-yeasts were isolated from two different sites: São Miguel dos Campos, Alagoas, Brazil, and Cosmópolis, São Paulo, Brazil. Samples from decayed energy cane and sugarcane and pest insects associated with this material were collected from the former location. Pest insects isolated from corn were isolated from the latter location. Decayed energy cane and sugarcane samples were collected closest to an ethanol mill, where the material was exposed to the environment for a long period. The local weather is hot and humid, with an average temperature from 20 to 32 °C and average annual precipitation of 1408 mm. Twenty samples of each material were collected in July 2015, stored in sterile recipients and transported under refrigeration to the laboratory in less than 24 h, according to Cadete et al. [76]. The same procedure was adopted for plague insects collected from plant material. For sugarcane and energy cane, 1 g of each sample was placed separately in 125 mL erlenmeyer flasks containing 50 mL of isolation xylose medium. For insects’ samples, guts were removed in a sterile environment and macerated with a micropistil in a 1.5 mL conical tube containing 400 µL of isolation medium, and then re-suspended separately in 125 mL erlenmeyer flasks containing 50 mL of isolation xylose medium. The flasks were incubated for 2–6 days in orbital shakers at 27 °C, 150 rpm until growth was visually detected. Five consecutive batches were carried out in the same medium to promote population enrichment and selection of strains capable of assimilating xylose rapidly. After growth, 50 µL of each medium was spread with Drigalski spatula in Petri dishes containing xylose isolation solid medium. Plates were incubated at 30 °C until colonies were observed. The isolated yeasts were maintained in permanent frozen stocks using 30% glycerol solution at − 80 °C [75].

### DNA extraction and molecular identification

C5-yeast species were identified using the internal transcribed spacer sequence (ITS1-5.8S-ITS2) and D1/D2 domains. DNA extraction was performed using the standard *S. cerevisiae* protocol described in Ausubel [75]. The ITS1-5.8S-ITS2 and D1/D2 sequences were amplified by PCR using GoTaq^®^ DNA polymerase (Promega) as described in FELL et al. [80] and Cadete et al. [76]. PCR amplicons were sequenced in a Sanger platform using an Applied Biosystems^®^ Genetic Analyzer 3500. The amplicons were manually trimmed using a base QV of 20 and grouped in contigs. These contigs were used in BLASTn megablast program at the NCBI database (http://blast.ncbi.nlm.nih.gov/Blast.cgi). The ITS and D1/D2 domains were retrieved from type material (CBS—http://www.cbs.knaw.nl/) of the top five hits in BLASTn and the sequences were globally aligned against the used query utilizing Clustal W in MEGA version 6 [81]. The query that had < 1% divergence to material type sequence, was considered from the same species of the type strain [82].

### Comparative fermentation of C5-yeasts

In order to select the most suitable source of genes related to xylose metabolism, yeasts isolated and molecularly identified were compared in YPX medium. The ability to ferment d-xylose was screened using Durham tubes and the yeast *S. stipitis* NRRL Y-7124 as a positive control. Tubes were incubated at 30 °C on shaker at 150 rpm for 10 days to observe gas production. The ability to assimilate xylose of the isolated yeast species was also compared in erlenmeyer with 150 mL of YPX (30 g/L) in orbital shakers, incubated at 30 °C and 150 rpm, starting with an optical density at 600 nm of approximately 1.0. All experiments were conducted in triplicates and samples were collected to measure optical density and to quantify metabolites with HPLC.

### Analytical procedures

Samples from fermentation were analyzed regarding glucose, xylose, ethanol, glycerol, xylitol, succinic acid and acetic acid concentrations using a Waters e2795 HPLC with an Hpx 84 h column (Biorad) A standard curve with known concentrations of the compounds highlighted above was also analyzed using the same procedure. Ethanol, xylitol and glycerol yields (Yp/s, g/g) were calculated considering the ratio of the specific product formation to sugar consumption. Productivity (Qp, g/L h) was calculated as the ratio of product concentration (g/L) and fermentation time (h).

### Phylogenetic analysis

The ability to ferment xylose for yeast species used in the phylogenetic analysis was assumed using the description of type strains deposited in Centraalbureau voor Schimmelcultures (CBS—http://www.cbs.knaw.nl/) yeast collection. We chose and downloaded a wide phylogenetic range of public yeast species genomes in Joint Genome Institute (JGI) Mycocosm from https://mycocosm.jgi.doe.gov/mycocosm/species-tree/tree;FrDK5q?organism=ascomycota. A large comparative analysis of these genomes was done by our group previously [37]. From the previous analysis, homologous gene families appeared. Orthology prediction was made using Markov clustering as implemented in OrthoMCL [83] which clusters orthologs and paralogs per similarity in functional groups called hereafter as gene families or orthogroups. The distribution of these families obeyed a numerical descending order, where larger families come before smaller families, being Fam0 the largest one. Transporter gene families were obtained using known xylose transporter proteins as baits in a BLAST search against gene families (Additional file 9: Table S5).

Assuming that all putative transporter gene families are homolog as a group, we proceeded with a gene phylogenetic tree reconstruction using all families and some outgroup sequences from bacteria and plants (Fig. 1). Amino acids sequences were globally aligned using MAFFT [84]. A maximum likelihood tree inference was carried in IQTree using its own adjustment substitution model inference (BIC and AIC criteria implemented) and 1000 bootstraps for branch support.

### Plasmid assembly

Expression cassettes and plasmids were assembled in a single reaction using the Gibson Assembly^®^ Master Mix (New England Biolabs—NEB) [85], and the backbone pRS426 [86] cleaved with *Bam*HI (Promega). The sequences coding the putative transporters were amplified by PCR using Phusion DNA polymerase (New England BioLabs—NEB) from the gDNA of *C. sojae*. The control transporter *GXF1* was amplified from *Candida intermedia* genome, using specific primers for each gene (Additional file 10: Table S6). Sequences of *TDH1* promoter and terminator were amplified from *S. cerevisiae* strain LVA1 genome [10]. Fragments from PCR amplifications were purified from agarose gel using the Kit Wizard^®^ SV Gel and PCR Clean-Up System (Promega) before the assembly reaction. Cassettes containing the genes *XYL1* and *XYL2* from *S. stipitis* and *XKS1* from *S. cerevisiae* were amplified from the plasmids pSsXRXDH and pScXKS (Santos, personal communication). Both cassettes use the *URA3* gene flanked by two *loxP* sites as marker. The correct frame of the gene sequences cloned in the plasmids and expression cassettes were confirmed by Sanger sequencing. The main components of the plasmids are summarized in Additional file 11: Table S7.

### Construction of *Saccharomyces cerevisiae* strains

The molecular techniques were performed using standard procedures [75]. The *S. cerevisiae* strains, primers and plasmids used in this study are listed in Additional files 4, 10, 11 Tables S2, S6 and S7, respectively. Expression cassettes containing the genes *XYL1*, *XYL2* and *XKS1* were amplified by PCR, purified from agarose gel using the kit Wizard^®^ SV Gel and PCR Clean-Up System (Promega) and used to transform EBY.VW4000 strain using the LiAc/SS-DNA/PEG protocol [87]. The stable integration of the two expression cassettes was molecularly verified. The auxotrophic marker *URA3* was excised using the plasmid pSH65 (Cre-loxP system) [79]. The Cre-recombinase enzyme, used to restore uracil auxotrophy, was induced when strains were grown in maltose. Counter-selection was performed using 5-FOA to select strains that excised *URA3*. Transformants of EBY_Xyl1 harboring the plasmids pCIGXF1, pCS186, pCS2608, pCS3894, pCS4130 and pRS426 were selected in YNB medium lacking uracil. The transformation was confirmed by PCR. Deletion of the hexokinase genes *HXK1*, *HXK2* and *GLK1* was carried out by insertion of the antibiotic markers hph, *KanMX4* and ble, amplified from pAG32 [78], pFA6KANMX4 [77] and pSH65 [79], respectively, resulting in EBY_Xyl1_*hxk*^*0*^ mutant cell (Additional file 4: Table S2).

### Microscopy of GFP-tagged strains

Codon-optimized monomeric Green Fluorescent Protein—GFP mUkG1 from soft coral [88] was synthesized by Genscript and fused to the transporter sequence in the plasmids. Strain EBY_Xyl1 harboring the Cs4130-mUkG1 was grown on YNB without uracil, supplemented with maltose 20 g/L overnight at 30 °C/200 rpm. Yeast cells were examined by confocal fluorescence microscopy (Leica TCS SP8 inverted microscope). The GFP emitted fluorescence was captured by a Hybrid Detector set to the *λ*-range of 503–530 nm. Differential Interference Contrast (DIC) images were also captured to show yeast morphology. Data were analyzed in LAS X core software (Leica microsystems).

### Spot growth assay

The growth of the EBY_Xyl1 mutant strains were evaluated by spot assay as previously described [31]. The yeast cells were cultured in YNB without uracil, supplemented with maltose 20 g/L for 24 h at 30 °C/200 rpm. Then, the cells were harvested by centrifugation at 3000 rpm for 5 min, washed three times and resuspended in water to OD_600_  =  1.0. Using an automatic multichannel pipette, 5 μL of a tenfold serial dilution of the cells were spotted on Petri dishes with YNB synthetic complete medium without uracil and different carbon sources (20 g/L: maltose (control), glucose, galactose, xylose, mannose or fructose). The growth of EBY_Xyl1 strain was also evaluated in YNB containing 5, 10, 30 and 50 g/L of xylose in order to evaluate if the strain growth is sugar concentration-dependent. The cells were incubated at 30 °C for 3 days (maltose), 5 days (glucose, galactose, mannose and fructose) and 7 days (xylose). All the experiments were performed in triplicate and repeated at least twice. For EBY_Xyl1_*hxk*^*0*^ drop assay, the cells were inoculated in 50 mL erlenmeyer flasks with YNB medium without uracil, supplement with 3% ethanol for 56 h at 30 °C/200 rpm. YNB was supplemented with 10 g/L of xylose and glucose (1:1), 10 g/L of xylose and 20 g/L of glucose (1:2) and 10 g/L of xylose and 50 g/L of glucose (1:5). Strains grown in YNB containing glucose 20 g/L, ethanol 3% or xylose 10 g/L were adopted as controls. All experiments were performed in triplicate and the cultures were incubated for 10 days.

### Fermentation assays

In order to confirm the Cs4130 behavior in different concentrations of sugar, fermentations were conducted in liquid media. The yeast strains were pre-grown on YNB synthetic medium (without uracil) supplemented with 5 g/L of casamino acids and 50 g/L of d-maltose until reach the stationary phase. Then, the cells were harvested by centrifugation, washed three times with sterile water and resuspended to an OD_600_ of 20. The fermentation experiments were performed using 60 mL of YNB supplemented with 5 g/L of casamino acids, 10 g/L and 50 g/L of xylose. For simultaneous glucose and xylose co-fermentation, 10 g/L of both sugars were used. The cells were incubated at 30 °C/200 rpm. The experiments were performed in triplicate and samples were collected to measure optical density and for HPLC analysis.

### Docking and normal mode analysis

Molecular Docking calculations were performed using UCSF Dock 6.9 [89]. The input files were prepared with UCSF Chimera 1.13.1 [90]. Cs4130 and Gxf1 tridimensional structures were modeled in I-TASSER web server [42]. The models were constructed based on the crystallographic structure of XylE (PBD code: 4GBY) complexed with d-xylose (TM-Score = 0.774), which indicates the most suitable regions for docking runs. 4GBY was employed as a reference to define the best force-field for sugar interactions based on previous reports of the influence of XylE variants for xylose transport [43]. The ligands were extracted from HIC-Up database (http://xray.bmc.uu.se/hicup/). Receptors and ligands were prepared using Dock Prep tool and the potential binding pockets were detected with Sphgen tool. Then, the closest cavities to d-xylose in XylE were chosen. Charges were added through UCSF Chimera using AMBER ss12sb and AM1-BCC force-fields for standard and non-standard residues, respectively. The molecular docking was carried out through Anchor and Grow algorithm and the energy of interaction was calculated with GridScore function due to the best agreement with the XylE mutants’ experimental results. Ligand, receptor, ligands orientation and overlap bins were set to 0.2 Å and the distance form matching among the atoms and receptor were set to 0.75 Å. The number of conformations evaluated per run (a total of 300 independent runs) was 500. The conformation with the lowest energy interaction value was selected. The effect of mutations in Cs4130 and Gxf1 were evaluated by two independent methods: Molecular Docking calculations and normal mode analysis (NMA). Residues close to xylose (< 5 Å) were evaluated through docking analysis since they may be directly related to transport. NMA otherwise can quickly and extensively evaluate how mutations could affect the protein fluctuation regime or indicate allosteric effects. Non-linear NMA were performed with NOLB software [44] by generating 20 samples and evaluating the 10 lowest frequencies through root-mean square fluctuation (RMSF) using GROMACS toolkit [91].

## Supplementary information

**Additional file 1: Figure S1.** Microbiomes exploited for the isolation of wild C5-yeasts. A. Termite associated with lignocellulosic material; B. Vessel formed by insect pest in energy cane; C. Sugarcane in a state of decomposition; D. Coleoptera larvae isolated from energy cane.

**Additional file 2: Table S1.** Fermentation performance of isolated C5 yeast species in YPX 20 g/L. Yield (Yp/s) is expressed as grams of product (gp) per grams of xylose. Xylose consumption was measured after 54 h of fermentation. C5 yeasts isolated from decayed energy cane (EC), sugarcane (SC), sugarcane straw (SS), corn crops (CC) and associated insect pests.

**Additional file 3: Figure S2.** *C. sojae* fermentation of xylose as the sole carbon source. *C. sojae* were cultivated in YPX (30 g/L) in batch fermentation with a low initial optical density of 0.5. Fermentation assays were performed in triplicate and error bars represent the standard deviation from the average values.

**Additional file 4: Table S2.** *S. cerevisiae* strains used in the study.

**Additional file 5: Table S3.** Variants of XylE [43] and equivalent residues in Cs4130, Gxf1, STP10 [92] and GlcP_SE_ [93].

**Additional file 6: Table S4.** Binding energies (kcal/mol) of Cs4130, Gxf1 mutants complexed with xylose through molecular docking analysis.

**Additional file 7: Figure S3.** Zoom inset of the main amino acids in Cs4130 (32-41, 257-263 and 515-529) with the dynamical regimes affected by the mutation R365A.

**Additional file 8: Figure S4.** Evaluation of the dynamical regimes of Cs4130 and Gxf1 through non-linear Normal Mode Analysis. The average of all low-frequencies for Cs4130 and Gxf1 is presented in A and B, respectively. The displacement of the residues was evaluated using the root mean square fluctuation (RMSF).

**Additional file 9: Table S5.** Xylose transporter proteins used in phylogenetic analysis.

**Additional file 10: Table S6.** Primers used in this study. Homology to promoter (Pr) and terminator (Ter) TDH1 are shown in bold.

**Additional file 11: Table S7.** Plasmids used in the study.

## Data Availability

All data generated or analyzed during this study are included in this published article and its additional files.

## Abbreviations

2G: Second-generation
MFS: Major facilitator superfamily
XR: Xylose reductase
XDH: Xylitol dehydrogenase
XKS1: Xylulokinase
*HXK*: Hexokinase
5-FOA: 5-Fluoroorotic acid
ITS: Internal transcribed spacer
NMA: Normal mode analysis
RMSF: Root-mean square fluctuation
UPR: Unfolded protein response
