# Supplementary material for: Novel xylose transporter Cs4130 expands the sugar uptake repertoire in recombinant Saccharomyces cerevisiae strains at high xylose concentrations
Source: Biotechnol Biofuels. 2020 Aug 14;13:145. doi: 10.1186/s13068-020-01782-0 (PMC7427733; doi:10.1186/s13068-020-01782-0)
Supplement: Supplementary file 1 — Additional file 1: Figure S1. Microbiomes exploited for the isolation of wild C5-yeasts. A. Termite associated with lignocellulosic material; B. Vessel formed by insect pest in energy cane; C. Sugarcane in a state of decomposition; D. Coleoptera larvae isolated from energy cane. [file 13068_2020_1782_MOESM1_ESM.docx]

**Additional file**

**
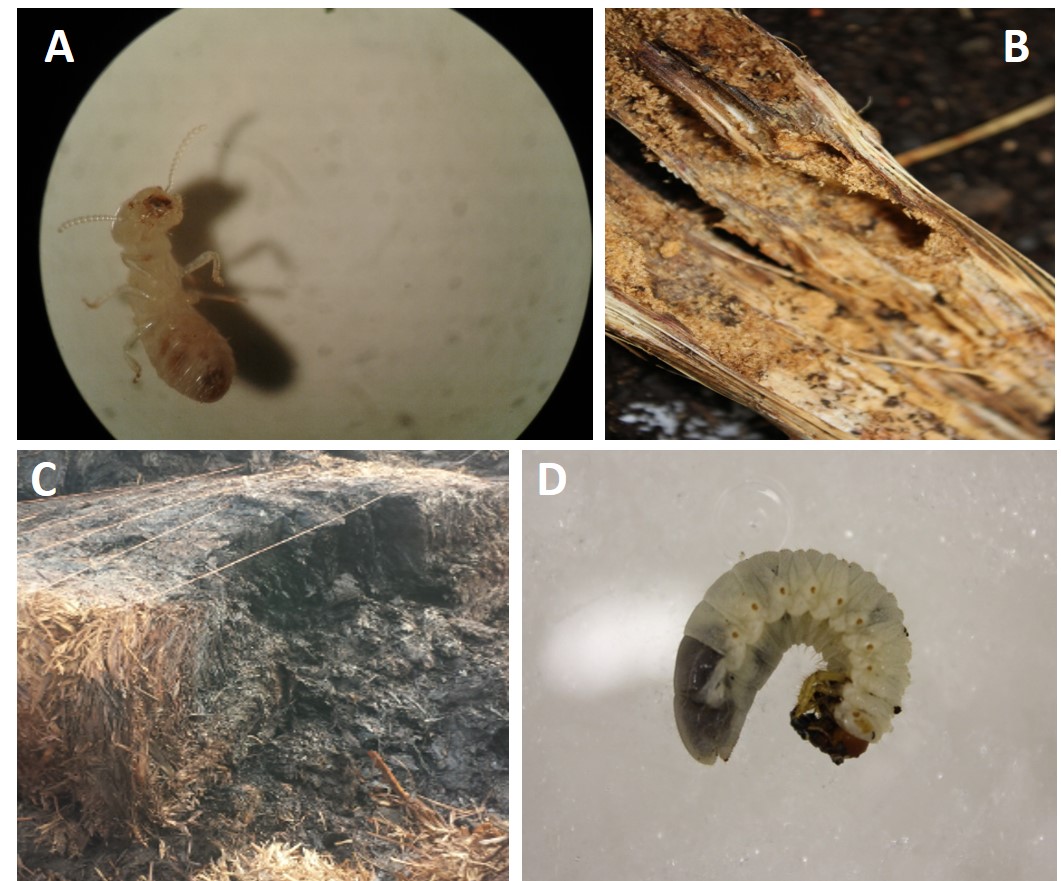
**

**Additional file 1: Figure S1. Microbiomes exploited for the isolation of wild C5-yeasts.** A. Termite associated with lignocellulosic material; B. Vessel formed by insect pest in energy cane; C. Sugarcane in a state of decomposition; D. Coleoptera larvae isolated from energy cane.
