# Supplementary material for: Novel xylose transporter Cs4130 expands the sugar uptake repertoire in recombinant Saccharomyces cerevisiae strains at high xylose concentrations
Source: Biotechnol Biofuels. 2020 Aug 14;13:145. doi: 10.1186/s13068-020-01782-0 (PMC7427733; doi:10.1186/s13068-020-01782-0)
Supplement: Supplementary file 2 — Additional file 2: Table S1. Fermentation performance of isolated C5 yeast species in YPX 20 g/L. Yield (Yp/s) is expressed as grams of product (gp) per grams of xylose. Xylose consumption was measured after 54 h of fermentation. C5 yeasts isolated from decayed energy cane (EC), sugarcane (SC), sugarcane straw (SS), corn crops (CC) and associated insect pests. [file 13068_2020_1782_MOESM2_ESM.docx]

**Additional file 2: Table S1.** Fermentation performance of isolated C5 yeast species in YPX 20 g/L. Yield (Yp/s) is expressed as grams of product (g_p_) per grams of xylose. Xylose consumption was measured after 54 hours of fermentation. C5 yeasts isolated from decayed energy cane (EC), sugarcane (SC), sugarcane straw (SS), corn crops (CC) and associated insect pests.

| **Yeast species** | **Source** | **Xylose consumed (%)** | **Yp/s^Xylitol^** | **Yp/s^EtOH^** | **Yp/s^Glycerol^** | |
| --- | --- | --- | --- | --- | --- | --- |
| *Blastobotrys adeninivorans* | EC | 66.5 | 0.11 | 0.12 | 0.15 |  |
| *Candida boidinii* | SS | 89.6 | 0.25 | 0.02 | 0.02 |  |
| *Candida pseudointermedia* | Coleoptera adult (CC) | 99.75 | 0.6 | 0.01 | 0.01 |  |
| *Candida sojae* | Coleoptera larvae (EC) | 100 | 0.67 | 0 | 0 |  |
| *Candida tropicalis* | Lepidoptera larvae (EC) | 100 | 0.6 | 0 | 0.02 |  |
| *Cryptococcus laurentii* | SC | 36 | 0.28 | 0.02 | 0.02 |  |
| *Kodameae ohmeri* | Hemiptera (CC) | 38.85 | 0.06 | 0 | 0 |  |
| *Meyerozyma caribbica* | EC | 68 | 0.3 | 0 | 0.01 |  |
| *Pichia sp.* | Coleoptera adult (SC) | 27.1 | 0.13 | 0.07 | 0.07 |  |
| *Pseudozyma sp.* | Hemiptera (CC) | 36.1 | 0.09 | 0.02 | 0 |  |
| *Trichosporon asahii* | SC | 36.75 | 0.28 | 0.02 | 0.01 |  |
| *Trichosporon coremeiformi* | Coleoptera adult (CC) | 28.5 | 0.11 | 0.03 | 0 |  |
| *Wickerhamomyces anomalus* | Coleoptera adult (SC) | 49.35 | 0.54 | 0.02 | 0 |  |
