# Supplementary material for: Novel xylose transporter Cs4130 expands the sugar uptake repertoire in recombinant Saccharomyces cerevisiae strains at high xylose concentrations
Source: Biotechnol Biofuels. 2020 Aug 14;13:145. doi: 10.1186/s13068-020-01782-0 (PMC7427733; doi:10.1186/s13068-020-01782-0)
Supplement: Supplementary file 3 — Additional file 3: Figure S2. C. sojae fermentation of xylose as the sole carbon source. C. sojae were cultivated in YPX (30 g/L) in batch fermentation with a low initial optical density of 0.5. Fermentation assays were performed in triplicate and error bars represent the standard deviation from the average values. [file 13068_2020_1782_MOESM3_ESM.docx]

**
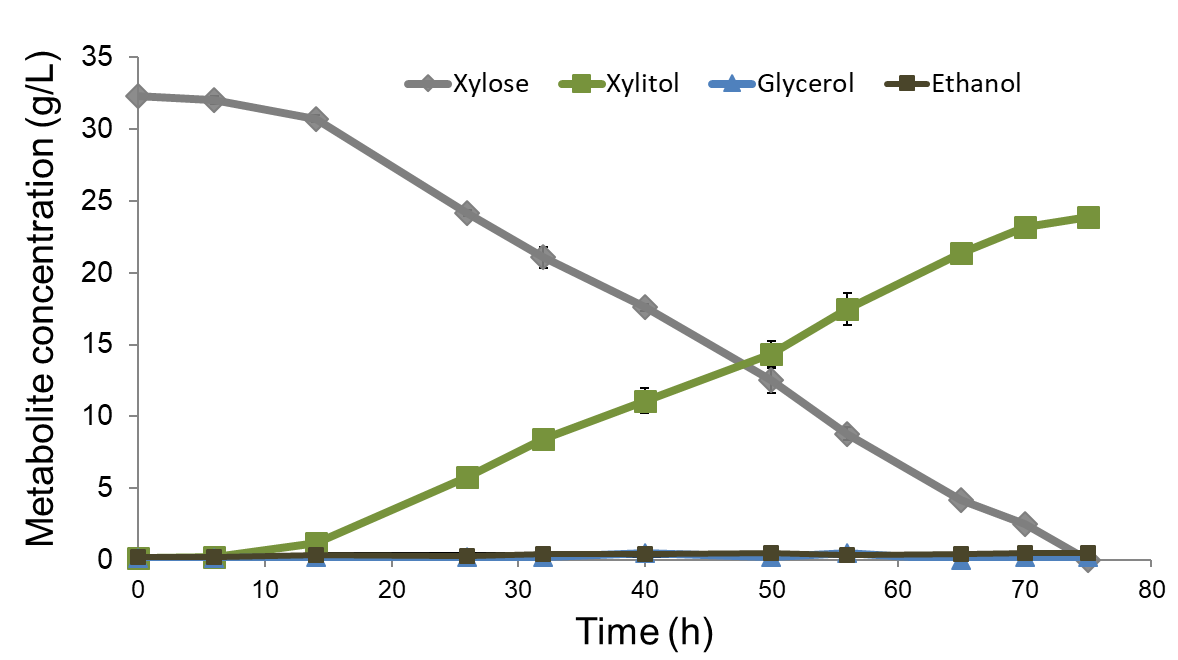
**

**Additional file 3: Figure S2. *C. sojae* fermentation of xylose as the sole carbon source.** *C. sojae* were cultivated in YPX (30 g/L) in batch fermentation with a low initial optical density of 0.5. Fermentation assays were performed in triplicate and error bars represent the standard deviation from the average values.
