# Supplementary material for: Novel xylose transporter Cs4130 expands the sugar uptake repertoire in recombinant Saccharomyces cerevisiae strains at high xylose concentrations
Source: Biotechnol Biofuels. 2020 Aug 14;13:145. doi: 10.1186/s13068-020-01782-0 (PMC7427733; doi:10.1186/s13068-020-01782-0)
Supplement: Supplementary file 4 — Additional file 4: Table S2. S. cerevisiae strains used in the study. [file 13068_2020_1782_MOESM4_ESM.docx]

**Supplementary material**

**Additional file 4: Table S2.** *S. cerevisiae* strains used in the study.

| **Strain** | **Relevant genotype/feature** | **Source** |
| --- | --- | --- |
| EBY.VW4000 | *CEN.PK2-1C hxt13Δ::loxP hxt15Δ::loxP hxt16Δ::loxP hxt14Δ::loxP hxt12Δ::loxP hxt9Δ::loxP hxt11Δ::loxP hxt10Δ::loxP hxt8Δ::loxP hxt514Δ::loxP hxt2Δ::loxP hxt367Δ::loxP gal2 Δ stl1Δ::loxP agt1Δ::loxP ydl247wΔ::loxP yjr160cΔ::loxP* | ^39^ |
|  |  |  |
| EBY_Xyl1 | *CEN5::pPGK1-XYL1-tPGK1-pTDH1-XYL2-tTDH1-pADH1-XKS1-tADH1* | This study |
|  |  |  |
| EBY_Xyl1_GXF1 | pCIGXF1 | This study |
| EBY_Xyl1_186 | pCS186 | This study |
| EBY_Xyl1_2608 | pCS2608 | This study |
| EBY_Xyl1_3894 | pCS3894 | This study |
| EBY_Xyl1_4130 | pCS4130 | This study |
| EBY_Xyl1_pRS | pRS426 | This study |
| EBY_Xyl1_hxk^0^ | *hphΔhxk1; kanΔhxk2; zeoΔglk1* | This study |
| EBY_Xyl1_ *hxk^0^*_4130 | pCS4130 | This study |
| EBY_Xyl1_ *hxk^0^*_GXF1 | pCIGXF1 | This study |
