# Supplementary material for: Novel xylose transporter Cs4130 expands the sugar uptake repertoire in recombinant Saccharomyces cerevisiae strains at high xylose concentrations
Source: Biotechnol Biofuels. 2020 Aug 14;13:145. doi: 10.1186/s13068-020-01782-0 (PMC7427733; doi:10.1186/s13068-020-01782-0)
Supplement: Supplementary file 5 — Additional file 5: Table S3. Variants of XylE [43] and equivalent residues in Cs4130, Gxf1, STP10 [92] and GlcPSE [93]. [file 13068_2020_1782_MOESM5_ESM.docx]

**Additional file 5: Table S3.** Variants of XylE [43] and equivalent residues in Cs4130, Gxf1, STP10 [92] and GlcP_SE_ [93].

| **XylE** | **Cs4130** | **Gxf1** | **STP10** | **GlcP_SE_** |
| --- | --- | --- | --- | --- |
| F24, decreases xylose transport | F58 | F58 | F39 | **Y19** |
| G83, abolishes xylose transport | G120 | G120 | G110 | G71 |
| R133, abolishes xylose transport | R153 | R153 | R142 | R102 |
| E153, abolishes xylose transport | E173 | E173 | E162 | E122 |
| R160, abolishes xylose transport | R180 | R180 | R169 | R129 |
| Q168, abolishes xylose transport | Q188 | Q188 | Q177 | Q137 |
| Q288, abolishes xylose transport | Q314 | Q314 | Q295 | Q250 |
| Q289, strongly decreases xylose transport | Q315 | Q315 | Q296 | Q251 |
| N294, abolishes xylose transport | N320 | N320 | N301 | N256 |
| Y298, abolishes xylose transport | Y324 | Y324 | **F305** | **F260** |
| N325, No effect in xylose transport | N349 | N349 | N332 | N287 |
| G340, abolishes xylose transport | G364 | G364 | G347 | **D302** |
| R341, abolishes xylose transport | R365 | R365 | R348 | R303 |
| W392, abolishes xylose transport | **Y428** | **Y431** | W410 | W357 |
| E397, abolishes xylose transport | E433 | E436 | E415 | E362 |
| R404, strongly decreases xylose transport | **K440** | R443 | R422 | R369 |
| Q415, strongly decreases xylose transport | **N451** | **N454** | **N433** | **L380** |
| W416, strongly decreases xylose transport | W452 | W455 | **M434** | **N381** |

The amino acids were mutated to alanine [55]. The only exception is 133R, which was mutated by C, H or L, but the phenotype was the same for all variants.

Substitutions of amino acids in the transporters, compared to XylE, are highlighted in bold.
