# Supplementary material for: Novel xylose transporter Cs4130 expands the sugar uptake repertoire in recombinant Saccharomyces cerevisiae strains at high xylose concentrations
Source: Biotechnol Biofuels. 2020 Aug 14;13:145. doi: 10.1186/s13068-020-01782-0 (PMC7427733; doi:10.1186/s13068-020-01782-0)
Supplement: Supplementary file 6 — Additional file 6: Table S4. Binding energies (kcal/mol) of Cs4130, Gxf1 mutants complexed with xylose through molecular docking analysis. [file 13068_2020_1782_MOESM6_ESM.docx]

**Additional file 6: Table S4.** Binding energies (kcal/mol) of Cs4130, Gxf1 mutants complexed with xylose through molecular docking analysis

| **GRID SCORE (kcal/mol)** | | | |
| --- | --- | --- | --- |
|  | **Cs4130** | **Gxf1** | **DtX (Å)** |
| **Wild-type** | -28.8 | -26.0 |  |
| **Mut 11** | I191A (-27.4/1.4) | I191A (-26.5/0.5) | 4.4 |
| **Mut 12** | I195A (-26.9/1.9) | I195A (-27.2/1.2) | 4.4 |
| **Mut 13**  **Mut 14** | F419A (-27.3/1.5)  Y428A (-28.5/0.3) | F422A (-25.5/0.5)  Y431A (-25.3/0.7) | 5.0  4.9 |

The energy of binding (kcal/mol) of mutants and the difference from the wild-type are

between parentheses.

Mut, mutant.
