# Supplementary material for: Novel xylose transporter Cs4130 expands the sugar uptake repertoire in recombinant Saccharomyces cerevisiae strains at high xylose concentrations
Source: Biotechnol Biofuels. 2020 Aug 14;13:145. doi: 10.1186/s13068-020-01782-0 (PMC7427733; doi:10.1186/s13068-020-01782-0)
Supplement: Supplementary file 7 — Additional file 7: Figure S3. Zoom inset of the main amino acids in Cs4130 (32-41, 257-263 and 515-529) with the dynamical regimes affected by the mutation R365A. [file 13068_2020_1782_MOESM7_ESM.docx]

**Supplementary material**


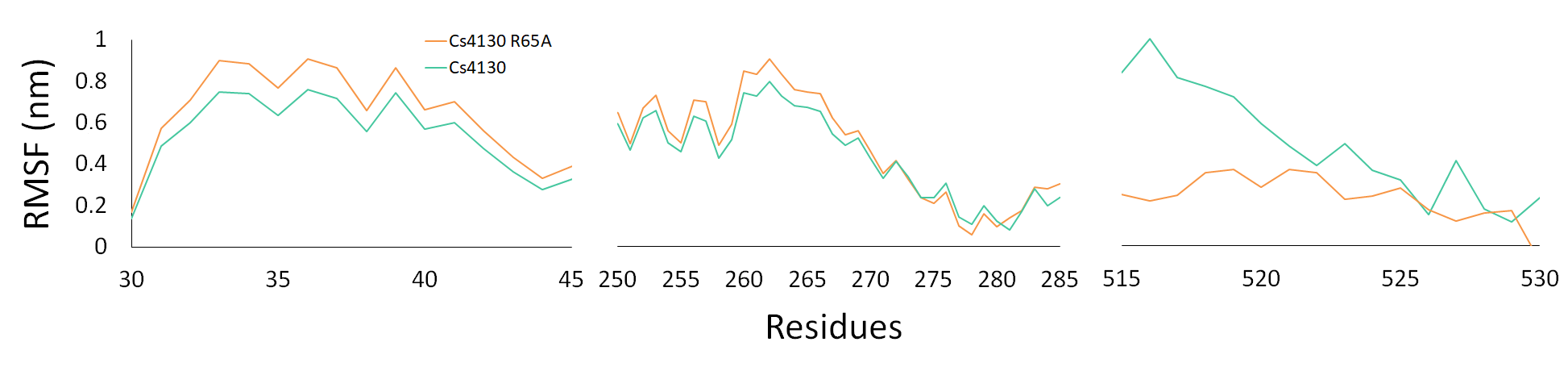


**Additional file 7: Figure S3.** Zoom inset of the main amino acids in Cs4130 (32-41, 257-263 and 515-529) with the dynamical regimes affected by the mutation R365A.
