# Supplementary material for: Novel xylose transporter Cs4130 expands the sugar uptake repertoire in recombinant Saccharomyces cerevisiae strains at high xylose concentrations
Source: Biotechnol Biofuels. 2020 Aug 14;13:145. doi: 10.1186/s13068-020-01782-0 (PMC7427733; doi:10.1186/s13068-020-01782-0)
Supplement: Supplementary file 8 — Additional file 8: Figure S4. Evaluation of the dynamical regimes of Cs4130 and Gxf1 through non-linear Normal Mode Analysis. The average of all low-frequencies for Cs4130 and Gxf1 is presented in A and B, respectively. The displacement of the residues was evaluated using the root mean square fluctuation (RMSF). [file 13068_2020_1782_MOESM8_ESM.docx]

**Supplementary material**

**
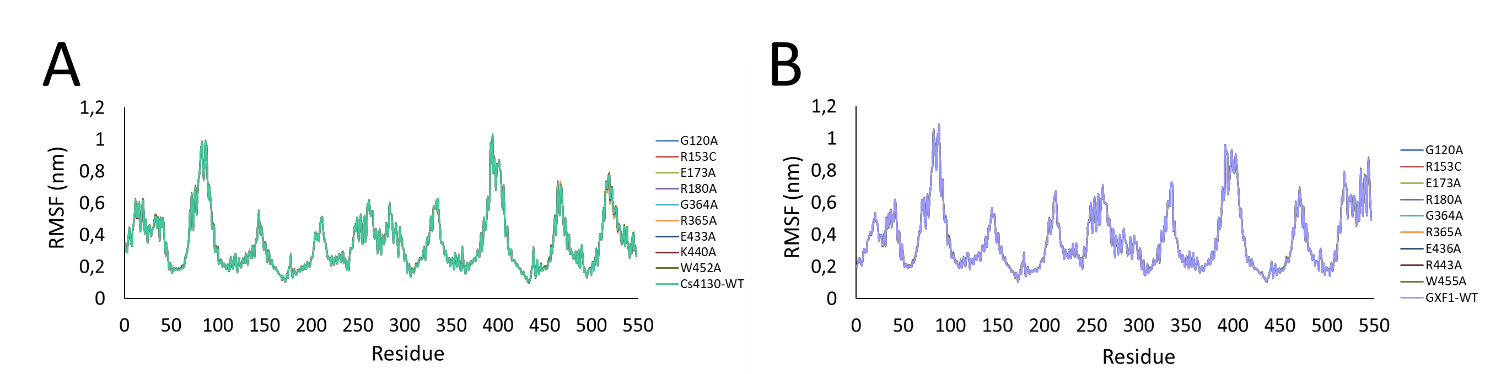
**

**Additional file 8: Figure S4.** Evaluation of the dynamical regimes of Cs4130 and Gxf1 through non-linear Normal Mode Analysis. The average of all low-frequencies for Cs4130 and Gxf1 is presented in A and B, respectively. The displacement of the residues was evaluated using the root mean square fluctuation (RMSF).
