# Supplementary material for: Novel xylose transporter Cs4130 expands the sugar uptake repertoire in recombinant Saccharomyces cerevisiae strains at high xylose concentrations
Source: Biotechnol Biofuels. 2020 Aug 14;13:145. doi: 10.1186/s13068-020-01782-0 (PMC7427733; doi:10.1186/s13068-020-01782-0)
Supplement: Supplementary file 9 — Additional file 9: Table S5. Xylose transporter proteins used in phylogenetic analysis. [file 13068_2020_1782_MOESM9_ESM.docx]

**Supplementary material**

**Additional file 9: Table S5.** Xylose transporter proteins used in phylogenetic analysis.

| **ID** | **Gene name** | **Accession** |
| --- | --- | --- |
| Q5A1D7 | *XUT1* | <https://www.uniprot.org/uniprot/Q5A1D7> |
| G4TIRP7 | *PIIN_05137* | <https://www.uniprot.org/uniprot/G4TIR7> |
| Q2MDH1 | *GXF1* | <https://www.uniprot.org/uniprot/Q2MDH1> |
| P32467 | *HXT4* | <https://www.uniprot.org/uniprot/P32467> |
| P39003 | *HXT6* | <https://www.uniprot.org/uniprot/P39003> |
| P39004 | *HXT7* | <https://www.uniprot.org/uniprot/P39004> |
| P13181 | *GAL2* | <https://www.uniprot.org/uniprot/P13181> |
| O07563 | *glcP* | <https://www.uniprot.org/uniprot/O07563> |
| Q8J0V1 | *mstA* | <https://www.uniprot.org/uniprot/Q8J0V1> |
| Q2MEV7 | *GXS1* | <https://www.uniprot.org/uniprot/Q2MEV7> |
| A3GHU5 | *XUT3* | <https://www.uniprot.org/uniprot/A3GHU5> |
| Q64L87 | *Xylhp* | <https://www.uniprot.org/uniprot/Q64L87> |
| Q5A1D7 | *XUT1* | <https://www.uniprot.org/uniprot/Q5A1D7> |
