# Supplementary material for: Novel xylose transporter Cs4130 expands the sugar uptake repertoire in recombinant Saccharomyces cerevisiae strains at high xylose concentrations
Source: Biotechnol Biofuels. 2020 Aug 14;13:145. doi: 10.1186/s13068-020-01782-0 (PMC7427733; doi:10.1186/s13068-020-01782-0)
Supplement: Supplementary file 10 — Additional file 10: Table S6. Primers used in this study. Homology to promoter (Pr) and terminator (Ter) TDH1 are shown in bold. [file 13068_2020_1782_MOESM10_ESM.docx]

**Supplementary material**

**Additional file 10: Table S6.** Primers used in this study. Homology to promoter (Pr) and terminator (Ter) *TDH1* are shown in bold.

| **Primer name** | **Sequence 5' to 3'** | **Description** |
| --- | --- | --- |
| Cs186_Forward | ATGCACGGTGGAGATTTAG | ORF amplification |
| Cs186_Reverse | TTATTCACTGATAACATCAGAATTTGTA |  |
| Cs186_Pr_Forward | **CACACACAAAAAACAGTACTTCACTAAATTTACACACAAAACAAA**ATGCACGGTGGAGATTTAG | pCS186 assemble |
| Cs186_Ter_Reverse | **AAATCATTATCCTCATCAAGATTGCTTTAT**TTATTCACTGATAACATCAGAATTTGTA |  |
| Cs2608_Forward | ATGGGTTTTAAAGATAGTGAATTGG | ORF amplification |
| Cs2608_Reverse | TTAGACTGAATCTTCTTCAACATAA |  |
| Cs2608_Pr_Forward | **CACACACAAAAAACAGTACTTCACTAAATTTACACACAAAACAAA**ATGGGTTTTAAAGATAGTGAATTGG | pCS2608 assemble |
| Cs2608_Ter_Reverse | **AAATCATTATCCTCATCAAGATTGCTTTA**TTTAGACTGAATCTTCTTCAACATAA |  |
| Cs3894_Forward | ATGAGTGCTACAGAACAAAATTCA | ORF amplification |
| Cs3894_Reverse | TTATGCAGATTCAGTTGCAGTT |  |
| Cs3894_Pr_Forward | **CACACACAAAAAACAGTACTTCACTAAATTTACACACAAAACAAA**ATGAGTGCTACAGAACAAAATTCA | pCS3894 assemble |
| Cs3894_Ter_Reverse | **AAATCATTATCCTCATCAAGATTGCTTTA**TTTATGCAGATTCAGTTGCAGTT |  |
| Cs4130_Forward | ATGTCTGTCTCCGACTC | ORF amplification |
| Cs4130_Reverse | CTAGACTTGTTCTTCATTTTGTTGT |  |
| Cs4130_Pr_Forward | **CACACACAAAAAACAGTACTTCACTAAATTTACACACAAAACAAA**ATGTCTGTCTCCGACTC | pCS4130 assemble |
| Cs4130_Ter_Reverse | **AAATCATTATCCTCATCAAGATTGCTTTAT**CTAGACTTGTTCTTCATTTTGTTG |  |
| ITS1_Forward | TCCGTAGGTGAACCTGCGG | Molecular identification |
| ITS4_Reverse | TCCTCCGCTTATTGATATGC |  |
