# Supplementary material for: Novel xylose transporter Cs4130 expands the sugar uptake repertoire in recombinant Saccharomyces cerevisiae strains at high xylose concentrations
Source: Biotechnol Biofuels. 2020 Aug 14;13:145. doi: 10.1186/s13068-020-01782-0 (PMC7427733; doi:10.1186/s13068-020-01782-0)
Supplement: Supplementary file 11 — Additional file 11: Table S7. Plasmids used in the study. [file 13068_2020_1782_MOESM11_ESM.docx]

**Supplementary material**

**Additional file 11: Table S7.** Plasmids used in the study

| **Plasmids** | **Parental** | **Relevant features** | **Source** |
| --- | --- | --- | --- |
| pRS426 |  | Multi copy plasmid, *URA3* | 86 |
| pSH65 |  | Cre recombinase, zeocin resistance | 79 |
| pRS304 |  | *TRP1* | 94 |
|  |  |  |  |
| pSsXRXDH | pRS304 | pPGK1-XYL1-tPGK1, loxP-URA3-loxP, pTDH1- XYL2-tTDH1 | Santos, personal communication |
| pScXKS1 |  | pADH1-XKS1-tADH1, loxP-URA3-loxP | Santos, personal communication |
|  |  |  |  |
| pCS186 | pRS426 | *pTDH1-Cs186-tTDH1* | This study |
| pCS2608 |  | *pTDH1-Cs2608-tTDH1* | This study |
| pCS3894 |  | *pTDH1-Cs3894-tTDH1* | This study |
| pCS4130 |  | *pTDH1-Cs4130-tTDH1* | This study |
| pCS4133 |  | *pTDH1-Cs4133-tTDH1* | This study |
| pCIGXF1 |  | *pTDH1-CIGXF1-tTDH1* | This study |
